# Targeting p53 for Melanoma Treatment: Counteracting Tumour Proliferation, Dissemination and Therapeutic Resistance

**DOI:** 10.3390/cancers13071648

**Published:** 2021-04-01

**Authors:** Joana B. Loureiro, Liliana Raimundo, Juliana Calheiros, Carla Carvalho, Valentina Barcherini, Nuno R. Lima, Célia Gomes, Maria Inês Almeida, Marco G. Alves, José Luís Costa, Maria M. M. Santos, Lucília Saraiva

**Affiliations:** 1 LAQV/REQUIMTE, Laboratόrio de Microbiologia, Departamento de Ciências Biolόgicas, Faculdade de Farmácia, Universidade do Porto, 4050-313 Porto, Portugal; up201407524@ff.up.pt (J.B.L.); liliana-raimundo@live.com (L.R.); julianameixedo@ua.pt (J.C.); pg32852@alunos.uminho.pt (C.C.); 2 Research Institute for Medicines (iMed.ULisboa), Faculty of Pharmacy, Universidade de Lisboa, 1649-003 Lisboa, Portugal; v.barcherini@campus.fct.unl.pt (V.B.); mariasantos@ff.ulisboa.pt (M.M.M.S.); 3 Coimbra Institute for Clinical and Biomedical Research (iCBR), Institute of Pharmacology and Experimental Therapeutics, Faculty of Medicine, University of Coimbra, 3000-548 Coimbra, Portugal; nuno.lima@uc.pt (N.R.L.); cgomes@fmed.uc.pt (C.G.); 4 Center for Innovative Biomedicine and Biotechnology (CIBB), University of Coimbra, 3000-548 Coimbra, Portugal; 5 Clinical Academic Center of Coimbra (CACC), 3000-548 Coimbra, Portugal; 6 ICBAS—Instituto de Ciências Biomédicas Abel Salazar, Universidade do Porto, Rua de Jorge Viterbo Ferreira 228, 4050-313 Porto, Portugal; ines.almeida@ineb.up.pt (M.I.A.); jcosta@ipatimup.pt (J.L.C.); 7 i3S—Instituto de Investigação e Inovação em Saúde, Universidade do Porto, Rua Alfredo Allen, 4200-135 Porto, Portugal; 8 INEB—Instituto de Engenharia Biomédica, Universidade do Porto, Rua Alfredo Allen, 4200-135 Porto, Portugal; 9 Department of Anatomy and Unit for Multidisciplinary Research in Biomedicine (UMIB), Institute of Biomedical Sciences Abel Salazar (ICBAS), University of Porto, 4050-313 Porto, Portugal; maalves@icbas.up.pt; 10 Institute of Molecular Pathology and Immunology of the University of Porto (IPATIMUP), 4200-135 Porto, Portugal; 11 Faculty of Medicine, University of Porto, Praça de Gomes Teixeira, 4099-002 Porto, Portugal

**Keywords:** melanoma, metastasis, drug resistance, targeted therapy, p53, tryptophanol-derived oxazoloisoindolinone

## Abstract

**Simple Summary:**

Melanoma is a highly metastatic and therapy-resistant cancer and is therefore associated with low survival rates of patients. In melanoma, the inactivation of the wild-type form of the p53 tumour suppressor protein is a frequent event, mainly through interactions with MDM2 and MDMX. In this work, our recently disclosed p53-activating agent, SLMP53-2, displayed promising in vitro and in vivo antitumour activity, with particular impacts on melanoma migration and invasion. Moreover, SLMP53-2 (re)sensitized melanoma cells to clinically used chemotherapeutic agents, potentially overcoming the therapeutic resistance issue. As a whole, the p53 activator SLMP53-2 may represent a new therapeutic opportunity for melanoma, particularly in combination with MAPK pathway-targeting drugs.

**Abstract:**

Melanoma is the deadliest form of skin cancer, primarily due to its high metastatic propensity and therapeutic resistance in advanced stages. The frequent inactivation of the p53 tumour suppressor protein in melanomagenesis may predict promising outcomes for p53 activators in melanoma therapy. Herein, we aimed to investigate the antitumor potential of the p53-activating agent SLMP53-2 against melanoma. Two- and three-dimensional cell cultures and xenograft mouse models were used to unveil the antitumor activity and the underlying molecular mechanism of SLMP53-2 in melanoma. SLMP53-2 inhibited the growth of human melanoma cells in a p53-dependent manner through induction of cell cycle arrest and apoptosis. Notably, SLMP53-2 induced p53 stabilization by disrupting the p53–MDM2 interaction, enhancing p53 transcriptional activity. It also promoted the expression of p53-regulated microRNAs (miRNAs), including miR-145 and miR-23a. Moreover, it displayed anti-invasive and antimigratory properties in melanoma cells by inhibiting the epithelial-to-mesenchymal transition (EMT), angiogenesis and extracellular lactate production. Importantly, SLMP53-2 did not induce resistance in melanoma cells. Additionally, it synergized with vemurafenib, dacarbazine and cisplatin, and resensitized vemurafenib-resistant cells. SLMP53-2 also exhibited antitumor activity in human melanoma xenograft mouse models by repressing cell proliferation and EMT while stimulating apoptosis. This work discloses the p53-activating agent SLMP53-2 which has promising therapeutic potential in advanced melanoma, either as a single agent or in combination therapy. By targeting p53, SLMP53-2 may counteract major features of melanoma aggressiveness.

## 1. Introduction

Skin cancer is among the most commonly occurring cancer type worldwide, particularly in the Caucasian population. Although only representing 5% of all skin cancers, melanoma is the most lethal subtype. This has been mainly attributed to its high metastatic potential and therapeutic resistance [1,2].

Melanoma is a highly heterogeneous tumour, comprising various genetic and molecular alterations [3]. Over recent years, the main driver mutations/alterations associated with melanoma have been described. In particular, the BRAFV600 mutation occurs in over 50% of melanoma cases (V600E substitution is the most frequent), leading to the constitutive activation of the mitogen-activated protein kinase (MAPK) pathway [4]. Accordingly, targeted therapies for melanoma, aiming BRAFV600E/K-expressing tumours, have involved the use of the BRAF inhibitor, vemurafenib, which was approved by the Food and Drug Administration (FDA) for unresectable or stage IV melanomas [5]. The efficacy of vemurafenib as a single agent was demonstrated with improved overall and progression-free survival in 50% of treated patients [6]. Nonetheless, acquired resistance to the clinically approved MAPK pathway-targeting drugs for melanoma, BRAF and mitogen-activated protein kinase kinase (MEK) inhibitors, have been frequently reported. As such, there are many expectations for forthcoming clinical trials investigating new drugs targeting different pathways to overcome chemoresistance in melanoma [7].

The p53 tumour suppressor protein is a major hub in a molecular network controlling numerous cellular processes, including proliferation, death and DNA repair [8,9]. Over recent years, the loss of p53 function has been closely associated with tumour formation, progression and dissemination [8,9]. In particular, it is known that p53 can regulate the expression of several genes involved in cell migration and invasion [10]. Based on this, it is expected that the restoration of p53 function will lead to tumour regression and metastasis prevention—namely, in melanoma [11]. In fact, the effect of a functional p53 in the abrogation of metastatic-related processes by melanoma cells has been demonstrated [12,13].

As a central player in carcinogenesis [9], p53 is to be found mutated in the majority of human cancers [14]. However, melanoma commonly harbours wild-type (wt) p53 (over 80–95% of melanoma cases; [15,16]). The low frequency of mutant (mut)p53 in melanoma has been mostly attributed to the inactivation of the cyclin-dependent kinase inhibitor 2A (CDKN2A) locus, encoding p16INK4A and p14ARF, which potentially renders the p53 mutation unwarranted. In fact, p14ARF directly inhibits mouse double minute 2 (MDM2), which is the major ubiquitin ligase involved in p53 degradation and inactivation. As such, under p14ARF deletion, p53 remains highly inhibited by MDM2 and unable to counteract tumour progression [7,17]. Notably, many chemotherapeutic agents have often proved to be ineffective due to an impairment of the p53 pathway. The combination of p53-activating agents, particularly inhibitors of the p53 interaction with MDM2 (e.g., nutlin-3a), with BRAF and MEK inhibitors, might therefore represent an appealing therapeutic strategy, potentially overcoming therapeutic resistance and improving disease-free survival of melanoma patients [7,18].

Recently, we disclosed the tryptophanol-derived oxazoloisoindolinone SLMP53-2 as a new p53-activating agent with in vitro and in vivo antitumor activity against hepatocellular carcinoma [19]. The p53-dependent antitumor activity of SLMP53-2 was demonstrated through re-establishment of the wt-like function to mutp53. This work also highlights the low toxicity of SLMP53-2 against normal cells and the absence of in vivo undesirable side effects.

Herein, we aimed to explore the antitumor potential of SLMP53-2, either as a single agent or in combination therapy in advanced melanoma. By targeting the p53 pathway, we intended to counteract major features of melanoma aggressiveness, particularly tumour dissemination and therapeutic resistance.

## 2. Results

### 2.1. SLMP53-2 Inhibits the Growth of Human Melanoma Cells Through Induction of Cell Cycle Arrest and Apoptosis

**Figure 1:**
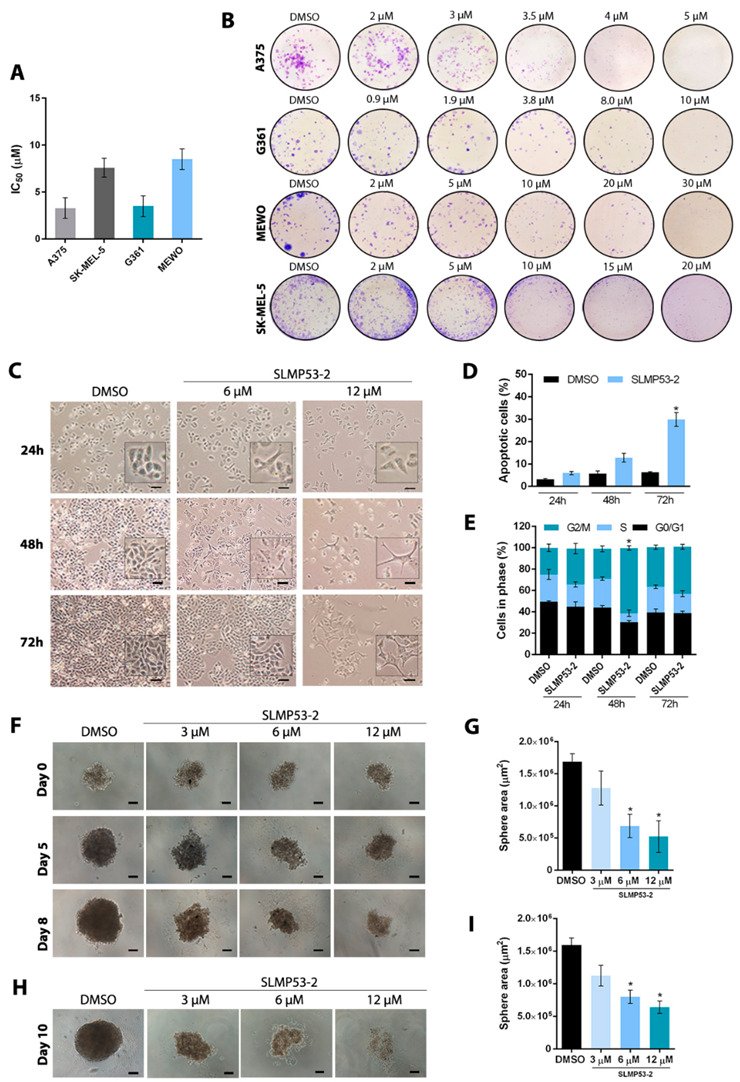
SLMP53-2 inhibits melanoma cell growth through induction of cell cycle arrest and apoptosis. (**A**) IC_50_ values of SLMP53-2 in A375, G361, MEWO and SK-MEL-5 melanoma cells obtained by colony formation assay for 11 days; data were normalized to DMSO and correspond to mean ± SEM, *n* = 5 (two replicates each). (**B**) Colony formation assay for A375, G361, MEWO and SK-MEL-5 melanoma cells treated with SLMP53-2 for the indicated concentrations. Images are representative of five independent experiments. (**C**) Effect of SLMP53-2 on growth and morphology of A375 cells for the indicated time points; images are representative of five independent experiments (scale bar  =  100 μm, magnification = ×100). (**D**) Apoptosis (Annexin V-positive cells) was evaluated in A375 cells after 24, 48 and 72 h of treatment with 12 μM SLMP53-2. (**E**) Cell cycle analysis in A375 cells was determined after 24, 48 and 72 h of treatment with 12 μM SLMP53-2. In (**D**,**E**), data are mean  ±  SEM, *n* = 5; values are significantly different from DMSO: * *p* < 0.05, one-way ANOVA followed by Tukey’s test. (**F**,**G**) Effect of SLMP53-2 on three-day-old A375 spheroids, for up to 8 days of treatment. In **G**, data are mean  ±  SEM, *n* = 5; values are significantly different from DMSO: * *p* < 0.05, one-way ANOVA followed by Tukey’s test. (**H**,**I**) Evaluation of spheroid formation after 10 days of treatment with SLMP53-2; treatment was performed at the seeding time of A375 cells. In **I**, data are mean  ±  SEM, *n* = 5; values are significantly different from DMSO: * *p* < 0.05, one-way ANOVA followed by Tukey’s test. In (**F**,**H**), images are representative of five independent experiments; scale bar = 100 μm; magnification = 100×.

In our previous work, the small-molecule SLMP53-2 was unveiled as a new activator of wt and mutp53 with promising antitumor activity, particularly in hepatocellular carcinoma [19]. Herein, the effectiveness of SLMP53-2 against cutaneous melanoma, which is a hard-to-treat tumour with a compromised p53 pathway, was investigated. To this end, the effect of SLMP53-2 on the proliferation and survival of melanoma cells expressing wtp53 (A375, SK-MEL-5, G361) or mutp53 (MEWO) (Figure S1) was assessed by colony formation assay. Using this cell survival assay, SLMP53-2 led to a 50% reduction in cell growth at 3.3 to 8.5 μM (Figure 1A,B).

For an in-depth analysis of the molecular mechanism underlying the antitumor activity of SLMP53-2 in melanoma cells, we focused on A375 cells. The A375 cell line was selected considering the promising antiproliferative activity of SLMP53-2 in these melanoma cells and its genetic background. In fact, the A375 cell line expresses p53 in its wt form, therefore being representative of most melanoma cells. Moreover, it expresses BRAF in its most frequent status in melanoma (mutBRAFV600E). The antiproliferative effect of SLMP53-2 on these cells was further evidenced by SRB assay (IC_50_ of 6.0 ± 1.0 μM, *n* = 6; Figure S2). This growth inhibition caused by SLMP53-2 in A375 cells was associated with changes in cell morphology (Figure 1C), induction of apoptosis, for 72 h (Figure 1D), and G2/M-phase cell cycle arrest for 48 h (Figure 1E) at 12 μM. The morphological changes observed in melanoma cells upon SLMP53-2 treatment, which caused the cells to resemble a dendritic-like shape, have been associated with a shift of melanoma cells to a morphology more resembling the differentiated status of their melanocyte precursors. This has also been related to a G2/M-phase cell cycle arrest and a loss of proliferative capacity. Indeed, melanocytes display differentiated “dendritic shapes” in healthy skin, differing from melanoma cell morphology. This differentiated morphology has been largely described for melanoma cells, namely those treated with antitumor agents [20,21,22].

The effect of SLMP53-2 was also evaluated on the growth and formation of a 3D spheroid model of A375 cells. A marked reduction in the area of three-day-old treated spheroids (Figure 1F,G; IC_50_ value of 5.5 ± 1.1 µM, *n* = 5) and generated (Figure 1H,I) spheroids was observed after 8 and 10 days of treatment, respectively, in particular at 6 and 12 μM of SLMP53-2.

Of note, we previously demonstrated that 6–18 µM of SLMP53-2 did not significantly interfere with the growth of nonmalignant cells (IC_50_ value of 50 μM, in fibroblast HFF-1 cells [19]).

### 2.2. SLMP53-2 Exerts a p53-Dependent Growth Inhibitory Effect through Enhancement of p53 Transcriptional Activity in Melanoma Cells

**Figure 2:**
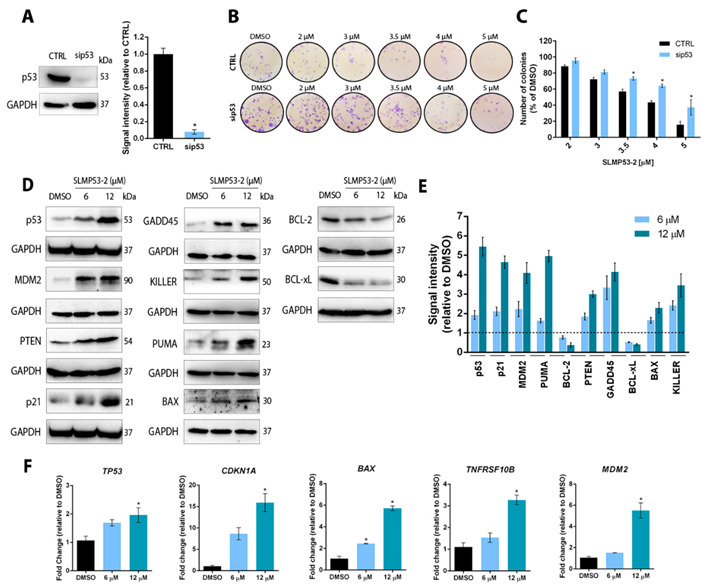
SLMP53-2 has p53-dependent growth inhibitory effect in melanoma cells with enhancement of p53 transcriptional activity. (**A**–**C**) Colony formation assay for silenced p53 (sip53) and control (CTRL) A375 cells treated with SLMP53-2, allowed to grow for 11 days. In (**A**), silencing efficacy of p53 by siRNA is shown; immunoblots are representative of five independent experiments and GAPDH was used as loading control; data plotted were normalized to CTRL and correspond to mean  ± SEM, *n* = 5; values are significantly different from CTRL: * *p* < 0.05, unpaired Student’s *t*-test. In (**B**), images are representative of five independent experiments. In (**C**), data are normalized to DMSO and correspond to mean ±  SEM, *n* = 5; values of sip53 cells significantly different from CTRL cells: * *p* < 0.05, two-way ANOVA followed by Sidak’s test. (**D**,**E**) Protein levels of p53 transcriptional targets in A375 cells treated with SLMP53-2 for 24 h (p53, MDM2, PTEN, Cyclin D1, p21 and KILLER) or 48 h (GADD45, PUMA, BCL-2, BCL-xL and BAX). In (**D**), immunoblots are representative of five independent experiments; GAPDH was used as loading control. In (**E**), quantification of protein expression levels is shown; values with DMSO were set as 1; data are mean  ±  SEM, *n* = 5. (**F**) mRNA levels of p53 target genes were determined by RT-qPCR in A375 cells after 24 h treatment with SLMP53-2; fold change is relative to DMSO; data are mean  ±  SEM, *n* = 5; values are significantly different from DMSO: * *p* < 0.05, two-way ANOVA with Dunnett’s multiple comparison test.

To assess the dependence of SLMP53-2 growth inhibitory activity on p53, the colony formation assay was performed in p53 small interfering RNA (siRNA)-silenced A375 cells (Figure 2A). In these cells, the growth inhibitory effect of SLMP53-2 was significantly reduced at 3.5, 4 and 5 μM when compared to control siRNA cells (CTRL; Figure 2B,C).

In A375 cells, SLMP53-2 also enhanced p53 transcriptional activity by regulating the protein and mRNA levels of several p53 transcriptional targets. In fact, 6 and 12 μM SLMP53-2 upregulated the protein levels of p53, MDM2, PTEN, as well as of proteins involved in G2/M cell cycle arrest (p21 and GADD45) and apoptosis (PUMA, BAX and KILLER). In addition, it downregulated the levels of the antiapoptotic proteins BCL-2 and BCL-xL (Figure 2D,E). It must be highlighted that similar effects of SLMP53-2 on the protein levels of p53 transcriptional targets could also be observed in wtp53-expressing SK-MEL-5 and mutp53-expressing MEWO cells (Figure S3). By RT-qPCR, we further confirmed that SLMP53-2 upregulated the mRNA levels of the p53 target genes *TP53*, *CDKN1A* (p21), *BAX*, *TNFRSF10B (KILLER)* and *MDM2*, mainly at 12 μM (Figure 2F).

**Figure 3:**
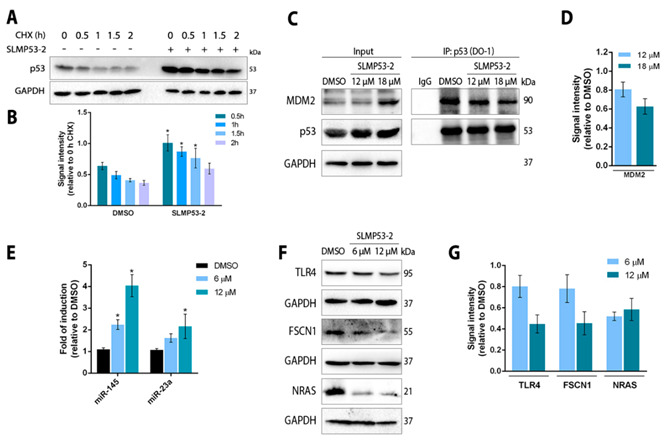
SLMP53-2 enhances p53 stabilization by disrupting the p53–MDM2 interaction and interferes with the miRNA network in melanoma cells. (**A**) p53 protein levels in A375 melanoma cells treated for 24 h with 12 µM SLMP53-2 or solvent followed by cycloheximide treatment from 0 to 2 h (CHX; 150 μg/mL). (**B**) Quantification of p53 protein expression levels; immunoblots are representative of five independent experiments; GAPDH was used as loading control. Values for cells nontreated with cycloheximide (0 h) were set as 1; data are mean  ±  SEM, *n* = 5. (**C**,**D**) Coimmunoprecipitation (Co-IP) was performed in A375 cells treated with SLMP53-2 for 4 h. In C, representative immunoblots of five independent experiments are shown—whole-cell lysate (Input). p53 from IP was used as loading control. In D, quantification of protein expression levels relative to DMSO is shown (set as 1). Data shown are mean ± SEM, *n* = 5. (**E**) Expression levels of miR-145 and miR-23a in A375 cells after 24 h of treatment with SLMP53-2 were determined by RT-qPCR; fold of change is relative to DMSO; data are mean  ±  SEM, *n* = 5; values are significantly different from DMSO: * *p* < 0.05, one-way ANOVA followed by Tukey’s test. (**F**,**G**) Protein levels of miR-145 target genes, in A375 cells treated with SLMP53-2 for 24 h. In (**F**), immunoblots are representative of five independent experiments; GAPDH was used as loading control. In (**G**), quantification of protein expression levels is shown; values with DMSO were set as 1; data are mean  ±  SEM, *n* = 5.

It was further verified that SLMP53-2 induced wtp53 stabilization. In fact, an enhancement of p53 half-life by SLMP53-2 was observed upon inhibition of protein synthesis with cycloheximide (Figure 3A,B). To further understand the mechanism underlying p53 stabilization, we started by assessing the ability of SLMP53-2 to promote the p53 interaction with heat shock proteins (Hsp), which may be involved in wtp53 stabilization, particularly Hsp70 and Hsp90 [23]. In fact, in mutp53-expressing hepatocellular carcinoma cells, SLMP53-2 restored wt-like conformation and transcriptional activity of mutp53 by promoting its interaction with Hsp70 [19]. However, by using Coimmunoprecipitation (Co-IP) analysis, we did not observe an enhancement of the Hsp70 or Hsp90 binding to wtp53 in melanoma (Figure S4). We next investigated whether SLMP53-2 could disrupt the p53 interaction with MDM2, a major p53 interactor involved in its inactivation and degradation by the ubiquitin–proteasome pathway [17]. For this, Co-IP analysis was performed on A375 cells treated with 12 and 18 µM SLMP53-2 for 4 h (Figure 3C,D). It should be noted that for such a short incubation time, the concentration of compound had to be increased to two- and three-fold the IC_50_ values. Notably, SLMP53-2 reduced the amount of MDM2 bound to p53, particularly at 18 µM, which evidenced an inhibition of the p53–MDM2 interaction by the compound.

The relevance of the miRNA network on melanoma pathogenesis led us to also assess the interference of SLMP53-2 on the levels of the tumour suppressors miR-145 and miR23a, which are direct targets of p53 regulation and crucial players in different melanomagenesis phases. The results showed that SLMP53-2 increased miR-145 and miR23a expression levels, particularly at 12 μM, in A375 cells (Figure 3E). Based on the pronounced enhancement observed for miR-145, the protein expression levels of its targets were also evaluated. Accordingly, 6 and 12 μM SLMP53-2 downregulated the protein levels of TLR4, FSCN1 and NRAS (Figure 3F,G).

### 2.3. SLMP53-2 Reduces Melanoma Cell Migration and Invasion

Considering metastization, the major cause of melanoma-related deaths, we investigated the potential of SLMP53-2 to prevent the migration and invasion of A375 and SK-MEL-5 melanoma cells. The SK-MEL-5 cells were also included in the study once obtained from a metastatic site of an axillary node. For further analysis with SK-MEL-5 cells, the IC_50_ of SLMP53-2 in this cell line was determined by SRB assay (9.5 ± 1.1 μM, *n* = 6; Figure S2).

**Figure 4:**
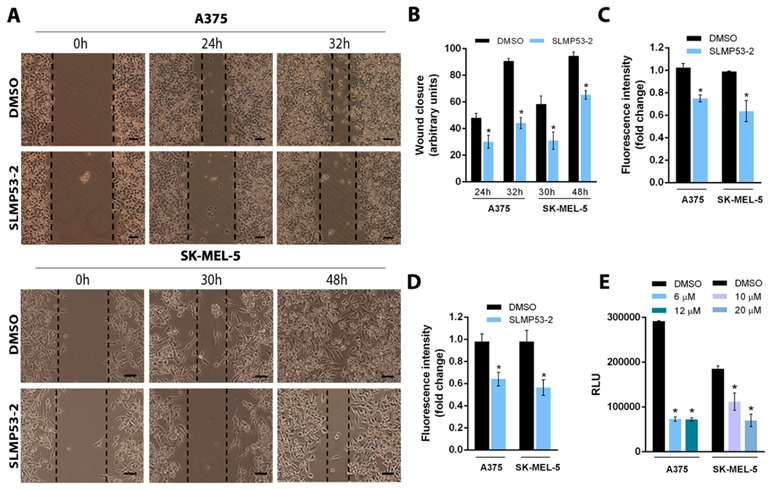
SLMP53-2 inhibits melanoma cell migration and invasion. (**A**) A375 and SK-MEL-5 confluent cells were treated with 2 or 4 μM SLMP53-2, respectively; cells were observed at 24 and 32 h (A375) and 30 and 48 h (SK-MEL-5) in the wound-healing assay. Images are representative of five independent experiments; scale bar = 100 μM; magnification = 100×. (**B**) Quantification of wound closure using randomly selected microscopic fields (six fields per sample). Data are mean ± SEM, *n* = 5; values are significantly different from DMSO: * *p* < 0.05, two-way ANOVA followed by Sidak’s test. (**C**) Effect of 2 μM SLMP53-2 on migration of A375 and SK-MEL-5 cells after 24 h of treatment. The relative number of migratory cells was determined by analysis of fluorescence signal intensity; values with DMSO were set as 1. Data are mean ± SEM, *n* = 5 (two replicates each); values are significantly different from DMSO: * *p* < 0.05, Student’s *t*-test. (**D**) Effect of 2 μM SLMP53-2 on the invasion of A375 and SK-MEL-5 cells after 24 h of treatment. Cells able to invade through an ECMatrix layer were quantified by fluorescence signal; values with DMSO were set as 1. Data are mean ± SEM, *n* = 5 (two replicates each); values are significantly different from DMSO: * *p* < 0.05, Student’s *t*-test. (**E**) Effect of SLMP53-2 on lactate secretion by A375 and SK-MEL-5 cells after 8 h of treatment. Cell density for each sample was used to normalize relative luminescence units (RLU) signal. Data are mean ± SEM, *n* = 5 (two replicates each); values are significantly different from DMSO: * *p* < 0.05; unpaired Student’s *t*-test.

The antimigratory activity of SLMP53-2 was firstly evaluated by a wound-healing assay. For the evaluated time points, 2 (in A375 cells) and 4 μM (in SK-MEL-5 cells) SLMP53-2 (concentrations with no significant effect on cell proliferation; Figure S2) significantly reduced wound closure (Figure 4A,B). Consistently, at the same concentrations, SLMP53-2 also inhibited the migration of A375 and SK-MEL-5 cells through a microporous membrane in the chemotaxis cell migration assay (Figure 4C), as well as the ability of these cells to invade through an ECMatrix layer (Figure 4D).

Considering that lactate secretion by tumour cells, with subsequent acidification of tumour microenvironment, is a well-known stimulating factor of their evasion [24,25], the levels of extracellular lactate were measured in melanoma cells treated with SLMP53-2. The results evidenced a marked reduction in extracellular lactate secreted by A375 (at 6 and 12 μM SLMP53-2) and SK-MEL-5 (at 10 and 20 μM SLMP53-2) cells (Figure 4E).

**Figure 5:**
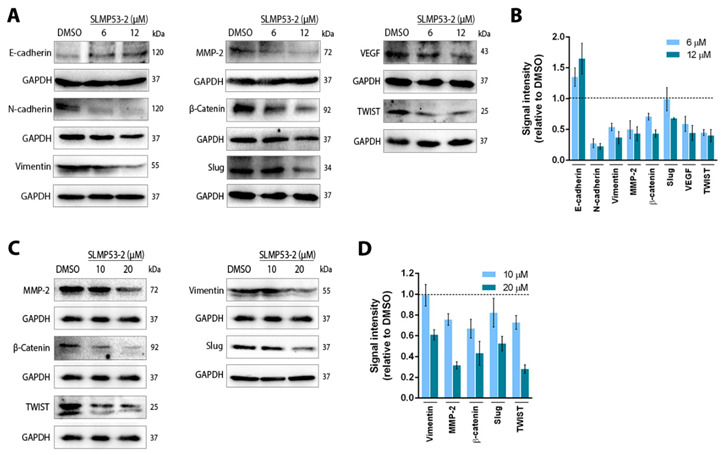
SLMP53-2 interferes with key molecular players in epithelial-to-mesenchymal transition (EMT) and angiogenesis. (**A**–**D**) Protein expression levels of crucial regulators of EMT and angiogenesis in A375 (**A**,**B**) and SK-MEL-5 (**C**,**D**) melanoma cells after 48 h of treatment with SLMP53-2 (in A375 cells, β-catenin was detected for 8 h and E-cadherin and TWIST for 24 h of treatment). Immunoblots are representative of five independent experiments; GAPDH was used as a loading control. In (**B**,**D**), quantification of protein expression levels is shown; values with DMSO were set as 1; data are means  ±  SEM, *n* = 5.

Accordingly, we also verified that SLMP53-2 inhibited epithelial-to-mesenchymal transition (EMT) markers in A375 and SK-MEL-5 cells. In fact, in A375 cells, 6–12 μM SLMP53-2 increased E-cadherin while decreasing N-cadherin, Vimentin, Slug, MMP-2, β-catenin and Twist protein levels (Figure 5A,B). Of note, a decrease in the angiogenic factor VEGF was also detected for 6–12 μM SLMP53-2 (Figure 5A,B). In SK-MEL-5 cells, 10–20 μM SLMP53-2 reduced the protein levels of MMP-2, β-catenin, Twist, Vimentin and Slug (Figure 5C,D).

### 2.4. SLMP53-2 Sensitizes Melanoma Cells to Clinically Available Chemotherapeutic Agents

**Figure 6:**
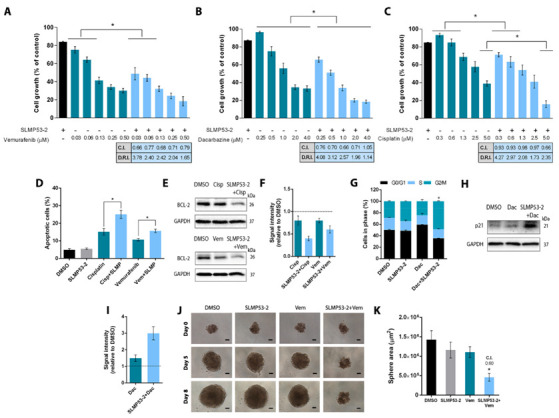
SLMP53-2 sensitizes melanoma cells to clinically used chemotherapeutic agents. (**A**–**C**) Cells were treated with a concentration range of vemurafenib (**A**), dacarbazine (**B**) and cisplatin (**C**) alone and in combination with 2 μM SLMP53-2, for 48 h, and the growth was analysed by SBR assay. Growth with DMSO was set as 100%. For each combination, the combination index (C.I.) and dose reduction index (D.R.I.) values were obtained. Data are mean  ±  SEM, *n* = 5 (two replicates each); values are significantly different from chemotherapeutic drug alone: * *p* < 0.05; two-way ANOVA followed by Sidak’s test. (**D**) Apoptosis (Annexin V-positive cells) was evaluated in A375 cells after 48 h of treatment with 2 μM SLMP53-2 (SLMP) and 5 µM cisplatin and 0.03 µM vemurafenib. Data are mean  ±  SEM, *n* = 5; values are significantly different from drug alone: * *p* < 0.05, one-way ANOVA followed by Tukey’s test. (**E**,**F**) Protein expression levels of BCL-2 after 48 h of treatment of SLMP53-2 with cisplatin (cisp) and with vemurafenib (vem). Immunoblots are representative of five independent experiments; GAPDH was used as a loading control. In **F**, quantification of protein expression levels; values with DMSO were set as 1; data are mean  ±  SEM, *n* = 5. (**G**) Cell cycle analysis in A375 cells was determined after 48 h of treatment with 2 μM SLMP53-2 and 2 μM dacarbazine (Dac). Data are mean  ±  SEM, *n* = 5; values are significantly different from drug alone: * *p* < 0.05, one-way ANOVA followed by Tukey’s test. (**H**,**I**) Protein expression levels of p21 after 48 h treatment of SLMP53-2 with dacarbazine (Dac). Immunoblots are representative of five independent experiments; GAPDH was used as a loading control. In **I**, quantification of protein expression levels is shown; values with DMSO were set as 1; data are mean  ±  SEM, *n* = 5. (**J**,**K**) Effect of 2 μM SLMP53-2 in combination with 0.027 μM Vemurafenib (Vem) on three-day-old A375 spheroids for up to 8 days of treatment. For the combination, the C.I. value was obtained. Images are representative of five independent experiments; scale bar = 100 μm; magnification = 100×. In (**K**), data are mean  ±  SEM, *n* = 5; values are significantly different from DMSO: * *p* < 0.05, one-way ANOVA followed by Tukey’s test.

The potential synergistic combination of SLMP53-2 with chemotherapeutic drugs currently used in melanoma therapy was assessed by SRB in A375 cells. For this, a concentration of SLMP53-2 with no significant effect on melanoma cell growth (2 μM) was tested with a range of concentrations of vemurafenib, dacarbazine and cisplatin (Figure 6A–C). The results showed that SLMP53-2 significantly increased the antiproliferative activity of these anticancer drugs when compared to their effects as single agents. Using the CompuSyn software, a multiple drug-effect analysis was performed for each combination and the combination index (C.I.) and dose reduction index (D.R.I.) values were calculated. Based on C.I. values, synergistic effects were obtained for most of the tested concentrations (C.I. < 1.0). The only exception occurred with cisplatin, which only synergised with SLMP53-2 at the highest tested concentration. The results also showed that the synergistic effects obtained for SLMP53-2 with cisplatin or vemurafenib were associated with increased apoptosis. In fact, for these combination regimens a visible increase in Annexin V-positive cells (Figure 6D), associated with a reduction of BCL-2 protein levels (Figure 6E,F) could be observed when compared to chemotherapeutics alone. However, when combined with dacarbazine, SLMP53-2 did not promote apoptosis. Instead, it induced cell cycle arrest at the G2/M phase (Figure 6G) and p21 expression (Figure 6H,I).

These results were further corroborated in a 3D spheroid model of A375 cells, in which we evaluated the combination of SLMP53-2 with vemurafenib (a BRAFV600E/K inhibitor commonly used in melanoma targeted therapy). In particular, 3-day-old spheroids were treated with 2 μM SLMP53-2 alone and in combination with 0.027 μM vemurafenib. As single agents, none of the compounds significantly interfered with melanoma spheroid growth at the tested concentrations (Figure 6J,K). However, in a combination regimen, a synergistic effect was achieved (C.I. of 0.60) with a marked reduction in the spheroid area (Figure 6J,K).

### 2.5. SLMP53-2 Does Not Induce Resistance in Melanoma Cells and Resensitizes Vemurafenib-Resistant Cells

**Figure 7:**
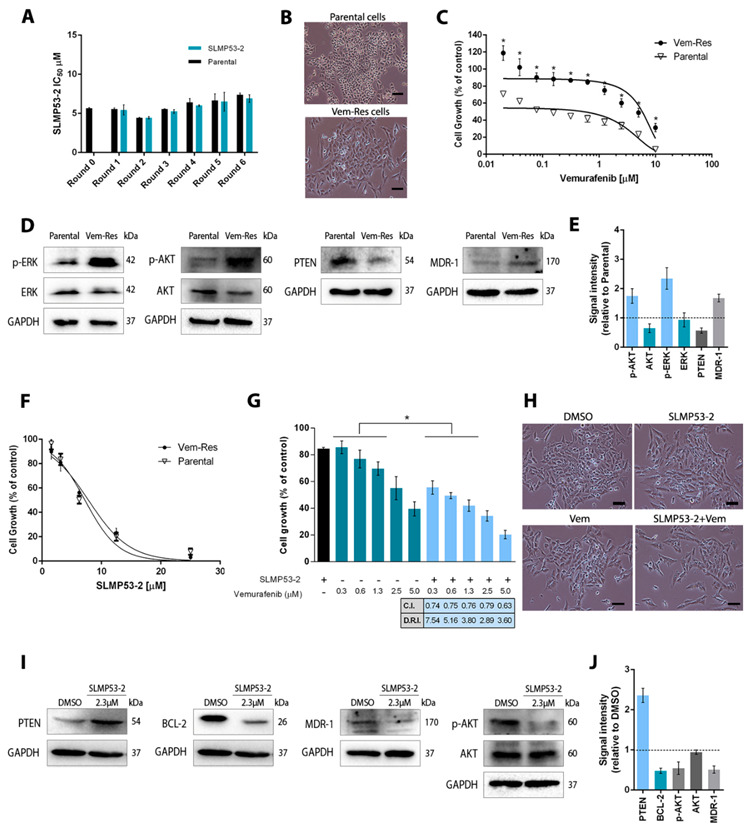
Melanoma cells do not develop resistance to SLMP53-2: vemurafenib-resistant melanoma cells show no cross-resistance to SLMP53-2 and are resensitized to vemurafenib by SLMP53-2. (**A**) A375 cells were exposed to six rounds of treatment with 6, 9, 12, 18, 24 and 30 μM of SLMP53-2. IC_50_ values were determined at the end of each round by SRB assay after 48 h of treatment. Data were normalized to DMSO and correspond to mean  ±  SEM, *n* = 5 (two replicates each); values not significantly different from parental cells: *p* > 0.05, two-way ANOVA followed by Sidak’s test. (**B**) Representative images of parental, vemurafenib-resistant (Vem-res) A375 cells; scale bar = 100 μm; magnification = 100×. (**C**) Concentration–response curves for vemurafenib in parental and Vem-res A375 cells after 48 h of treatment. Data were normalized to DMSO and correspond to mean  ±  SEM, *n* = 6 (two replicates each); values of Vem-res cells significantly different from parental cells: * *p* < 0.05; two-way ANOVA followed by Sidak’s test. (**D**,**E**) Protein levels of p-AKT/AKT, p-ERK/ERK, PTEN and MDR-1 in untreated parental and Vem-res A375 cells. In D, immunoblots are representative of five independent experiments; GAPDH was used as loading control. In E, quantification of protein expression levels is shown; values with DMSO were set as 1; data are mean  ±  SEM, *n* = 5. (**F**) Concentration–response curves for SLMP53-2 in parental and Vem-res A375 cells after 48 h of treatment. Data were normalized to DMSO and correspond to mean  ±  SEM, *n* = 6 (two replicates each); values of Vem-res cells are not significantly different from parental cells: two-way ANOVA followed by Sidak’s test. (**G**) Vem-res A375 cells were treated with a concentration range of vemurafenib alone and in combination with 2 μM of SLMP53-2. Cell growth was evaluated for 48 h of treatment; growth obtained with DMSO was set as 100%. For each combination, the C.I. and D.R.I. values were obtained. Data are mean ± SEM, *n* = 6 (two replicates each); values are significantly different from vemurafenib alone: * *p* < 0.05, two-way ANOVA followed by Sidak’s test. (**H**) Representative images of Vem-res A375 cells treated with DMSO, 2 μM SLMP53-2, 1.3 μM vemurafenib (Vem) and the combination (SLMP53-2 + Vem) for 48 h; images are representative of five treatments; scale bar = 100 μm; magnificatio*n* = 100×. (**I**,**J**) Protein levels of PTEN, BCL-2, MDR-1 and p-AKT/AKT, in Vem-res cells after 48 h of treatment with 2 µM SLMP53-2. In I, immunoblots are representative of five independent experiments; GAPDH was used as loading control. In J, quantification of protein expression levels is shown; values with DMSO were set as 1; data are mean  ±  SEM, *n* = 5.

The acquisition of resistance, particularly to BRAF inhibitors such as vemurafenib, remains one of the most reported drawbacks in melanoma targeted therapy [26]. To address this issue, we evaluated whether SLMP53-2 was able to induce resistance in A375 cells. After six rounds of treatment with increasing concentrations of SLMP53-2, melanoma cells did not develop resistance, as evidenced by the constant IC_50_ values of the compound in successive generations compared to parental cells (Figure 7A).

Since multiple drug resistance (MDR) is a common event in cancer cells, we next analysed whether vemurafenib-resistant (Vem-res) A375 cells could develop cross-resistance to SLMP53-2. For this, we established Vem-res A375 cells, which evidenced the lower antiproliferative effect of vemurafenib (IC_50_ of 4.3 ± 1.1 μM, *n* = 6) when compared to nontreated cells (parental; IC_50_ of 0.17 ± 0.02 μM, *n* = 6) (Figure 7B,C). It should be noted that although a longer treatment time was used to generate A375 cells with resistance to vemurafenib, when compared to SLMP53-2, the occurrence of a resistance phenotype could be observed from five rounds of treatment with vemurafenib (Figure S5).

To investigate the molecular mechanisms underlying the developed resistance of A375 cells to vemurafenib, major players associated with melanoma therapeutic resistance were investigated. Particularly, the reactivation of MAPK (leading to ERK activation) or PI3K-AKT pathways are commonly reported in melanoma patients resistant to MAPK inhibitors [26,27]. Exacerbated PI3K-AKT signalling can also occur due to loss of PTEN (a p53 transcriptional target and inhibitor of the PI3K-AKT pathway) in melanoma resistance to targeted therapy [11,27,28]. Several studies have also reported the overexpression of the multiple drug resistance transporter ABCB1 (MDR1) in resistant melanoma under treatment [29]. Based on this, the activation of ERK and AKT (by analysis of their phosphorylation levels) and the protein levels of PTEN and MDR1 were investigated, in Vem-res A375 cells. Accordingly, the established Vem-res A375 cells presented a partial loss of PTEN expression and increased protein levels of phosphorylated forms of ERK (p-ERK) and AKT (p-AKT), as well as of MDR1 (Figure 7D,E).

We next analysed the effect of SLMP53-2 on the growth of parental and Vem-res A375 cells. The results showed a similar sensitivity of both cells to SLMP53-2, which demonstrated that cross-resistance was not acquired by Vem-res A375 cells (Figure 7F). Importantly, we also verified that SLMP53-2 led to a resensitization of Vem-res A375 cells to vemurafenib. In fact, the combination of 2 μM SLMP53-2 with a concentration range of vemurafenib resulted in a greater growth inhibitory effect than vemurafenib alone (Figure 7G,H). Indeed, the synergistic effect between SLMP53-2 and vemurafenib could be evidenced by a C.I. < 1.0 for all tested concentrations. Consistently, the D.R.I. values also revealed a notable reduction in the effective dose of vemurafenib by its combination with SLMP53-2 in Vem-res melanoma cells (Figure 7G).

Interestingly, the resensitization of Vem-res melanoma cells by 2 μM SLMP53-2 was associated with enhancement of PTEN and reduction in p-AKT and MDR1 protein levels (Figure 7I,J). Additionally, a downregulation of BCL-2 protein levels was also observed in Vem-res A375 cells treated with SLMP53-2 (Figure 7I,J). In fact, the transcriptional expression of BCL-2 is negatively regulated by p53, and its overexpression has been related to cell death evasion and drug resistance in melanoma [11].

### 2.6. SLMP53-2 Displays In Vivo Antitumour Activity against Melanoma

The antitumor potential of SLMP53-2 was evaluated in xenograft mouse models of A375 cells at 50 mg∙kg^−1^, which corresponds to the lowest concentration of compound that led to the maximum tumour growth inhibitory effect in our previous work [19]. Of note, no undesirable haematological and biochemical toxicity was induced by intraperitoneal administrations of 50 mg∙kg^−1^ SLMP53-2 in rats [19].

**Figure 8:**
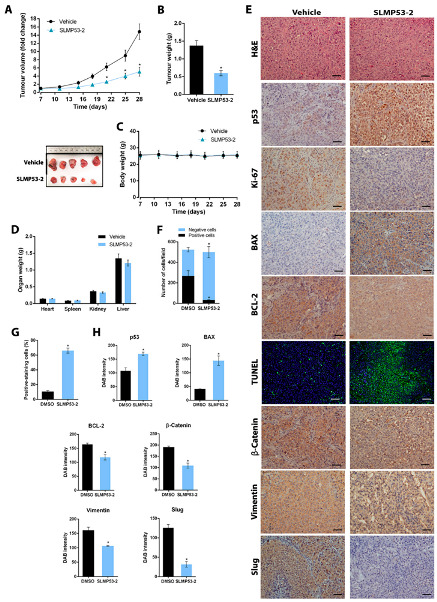
In vivo melanoma antitumour activity of SLMP53-2. C57BL/6-Rag2^−/−^IL2rg^−/−^ mice carrying A375 xenografts were treated with 50 mg∙kg^−1^ SLMP53-2 or vehicle by intraperitoneal injection twice a week for a total of six administrations. (**A**) Tumour volume curves of mice carrying A375 xenografts treated with SLMP53-2 or vehicle. Fold change is relative to the start of treatments; data are mean ± SEM, *n* = 7; values are significantly different from vehicle: * *p* < 0.05, two-way ANOVA followed by Sidak’s test. (**B**) Tumour weights measured at the end of the in vivo experiment; data are mean ± SEM, *n* = 7; values are significantly different from vehicle: * *p* < 0.05, unpaired Student’s *t*-test. Representative images of the tumours treated with SLMP53-2 or vehicle at the end of the experiment. (**C**) Body weight of the mice registered during the course of the experiment. Data are mean ± SEM, *n* = 7; values are not significantly different from vehicle: *p* > 0.05, two-way ANOVA followed by Sidak’s test. (**D**) Weight of heart, spleen, kidney and livers from animals treated with SLMP53-2 or vehicle. Data are mean ± SEM, *n* = 7; values are not significantly different from vehicle: *p* > 0.05, two-way ANOVA followed by Sidak’s test. (**E**) Representative images of p53, Ki-67, BAX, BCL-2, TUNEL, β-catenin, Vimentin, and Slug detection in tumour tissues of A375 xenografts treated with SLMP53-2 or vehicle, collected at the end of treatment (scale bar = 5 μm; magnificatio*n* = 200×); haematoxylin and eosin (H&E). (**F**–**H**) Quantification of immunohistochemistry of A375 xenograft tumour tissues treated with SLMP53-2 or vehicle. In F, quantification of the number of Ki-67-positive and -negative cells; values are significantly different from vehicle: * *p* < 0.05, two-way ANOVA followed by Sidak’s test. In G, quantification of the percentage of positive-staining cells with TUNEL, *n* = 5; values are significantly different from vehicle: * *p* < 0.05, unpaired Student’s *t*-test. In H, quantification of the p53, Vimentin, BAX, BCL-2, β-catenin and Slug staining by evaluation of 3,3′-diaminobenzidine (DAB) intensity is shown, *n* = 5; values are significantly different from vehicle: * *p* < 0.05, unpaired Student’s *t*-test.

Six intraperitoneal administrations of SLMP53-2 inhibited the growth of melanoma tumours when compared to vehicle (Figure 8A). Consistently, the weight of the collected tumours was significantly reduced in SLMP53-2-treated tumours (Figure 8B). Moreover, no significant body weight loss or morbidity signs were observed in SLMP53-2-treated mice compared to vehicle throughout the experiment (Figure 8C). Additionally, no significant differences were observed between the weight of heart, spleen, kidney and livers of SLMP53-2-treated mice and vehicle (Figure 8D).

The Immunohistochemical (IHC) staining of the tumour sections showed that, when compared to vehicle, SLMP53-2 decreased Ki-67, Vimentin, β-catenin, Slug and BCL-2, while increasing p53, BAX and TUNEL staining (Figure 8E–H). These results support an in vivo antitumor activity of SLMP53-2 through inhibition of cell proliferation and EMT and stimulation of apoptosis.

## 3. Discussion

Although not representing the most common type of skin cancer, melanoma is undoubtedly the most aggressive and deadly. One of the main features attributed to this type of tumour is the frequency of intrinsic or acquired resistance mechanisms [27]. In fact, despite the notable increase in overall survival of treated patients, a significant percentage of melanoma patients do not effectively respond to the available therapies. In most cases, the monotherapy has led to resistance scenarios that have not been settled by combining drugs directed to the MAPK pathway. Given the major role of the p53 tumour suppressor protein and its reported implication in MAPK-driven melanomas [30,31], the combined targeting of the MAPK and p53 signalling pathways has been highlighted as a promising therapeutic strategy for melanoma patients [7,32,33,34].

In our recent work, the small-molecule SLMP53-2 was disclosed as a new p53-activating agent able to restore wt-like function to mutp53 [19]. In that work, SLMP53-2 displayed potent growth inhibitory activity in hepatocellular carcinoma cells expressing either wt or mutp53.

In the present study, the antitumor potential of SLMP53-2 against melanoma cells was investigated, either as a single agent or in combination therapy. The tumour growth inhibitory effect of SLMP53-2 was confirmed in a panel of melanoma cell lines expressing wt or mutp53. The ability of SLMP53-2 to reduce proliferation of melanoma cells expressing wtp53 was also substantiated in 3D spheroid models of melanoma cells. Silencing of wtp53 in melanoma cells significantly decreased the tumour growth inhibitory activity of SLMP53-2, which further reinforced the p53-dependent antitumor activity of SLMP53-2 unveiled in our previous work [19]. In fact, also in hepatocellular carcinoma cells, the p53 knockout markedly reduced the mRNA and protein expression levels of several p53 transcriptional targets induced by SLMP53-2. Furthermore, SLMP53-2 re-established mutp53 DNA-binding ability [19]. Herein, we showed that SLMP53-2 induced wtp53 activation and stabilization through disruption of the p53 interaction with MDM2. As previously mentioned, since *TP53* mutations are rare in melanoma, activation of wtp53, by releasing it from the prominent MDM2 inhibitory effect, is considered a promising strategy in melanoma therapy [7,17,35].

In wtp53-expressing melanoma cells, SLMP53-2 consistently induced cell cycle arrest and apoptosis and markedly increased p53 transcriptional activity, as evidenced by the regulation of mRNA and protein expression levels of several p53 transcriptional targets. In particular, SLMP53-2 increased the levels of MDM2 and PTEN. Notably, PTEN is a tumour suppressor protein with a major role in cell proliferation, death, migration and adhesion, whose expression is found lost in approximately 20% of melanomas [36]. Moreover, in accordance with a G2/M-phase cell cycle arrest, SLMP53-2 increased the expression of p21 and GADD45. SLMP53-2 also regulated the expression levels of several p53 targets involved in apoptosis, upregulating PUMA, BAX and KILLER and downregulating the antiapoptotic proteins BCL-2 and BCL-xL.

Compelling evidence has shown that miRNAs are important regulators in melanoma progression and dissemination. Since miRNAs are frequently dysregulated in several types of cancer, they have been considered as effective therapeutic targets. From the numerous miRNAs dysregulated in melanoma, some are directly regulated by p53, including miR-145 and miR-23a, which are tumour suppressors with crucial roles in distinct phases of melanomagenesis [11]. In fact, these miRNAs are frequently downregulated in melanoma, which correlates with poor prognosis in melanoma patients. Accordingly, their expressions have been associated with improved long-term survival in metastatic melanoma patients and a significant reduction in cell proliferation, migration, and drug resistance [11]. In the present work, we unveiled that SLMP53-2 significantly increased the expression levels of miR-145 and miR-23a. In particular, the pronounced enhancement of miR-145 levels by SLMP53-2 was correlated with downregulation of its targets—TLR4, FSCN1 and NRAS. Particularly, it has been suggested that TLR4 may contribute to melanoma progression and migration [37] and that FSCN1 promotes EMT events [38]. Notably, in other works, this negative regulation of NRAS expression by miR-145 has been associated with inhibition of proliferation, invasion, and migration of melanoma cells [39].

Consistently, SLMP53-2 displayed potent anti-invasive and antimigratory properties in melanoma cells (including metastatic site-derived cells). Accordingly, the expression levels of relevant markers of EMT inhibition were evaluated in melanoma cells treated with SLMP53-2. EMT has been described as a crucial event in tumour dissemination, orchestrating alterations in the integrity of cell–cell junctions and cell–extracellular matrix, loss of polarity and epithelial markers (e.g., E-cadherin), which subsequently result in loss of contact between adjacent cells. As such, the acquisition of a more mesenchymal-like phenotype prompts cells to become more prone to migrating and so invade the nearby tissues [40,41]. In melanoma, throughout the radial growth phase (RGP), the interactions of melanoma cells with keratinocytes decrease, mainly due to the loss of E-cadherin expression. The successive vertical growth phase (VGP) is characterized by a transmigration of melanoma cells from the epidermis across the basal lamina to the dermis [42]. In accordance with the crucial role of p53 in suppressing major players of classical metastasis pathways, including cell adhesion, motility, invasion and EMT [10], SLMP53-2 increased E-cadherin and decreased N-cadherin, MMP-2 (relevant in degrading extracellular matrix components), Vimentin, β-catenin, Slug and Twist expression levels. It should be noted that despite controversial results regarding the role of β-catenin in melanoma dissemination, our results are in line with several reports sustaining a promigratory and proinvasive role of β-catenin in melanoma cells [43,44]. SLMP53-2 also decreased the levels of the angiogenesis-inducing factor VEGF and markedly reduced the lactate levels secreted to the cell medium. Actually, the lactate secretion from tumour cells (due to metabolic changes) favours a low pH in the microenvironment associated with an enhancement of angiogenesis through upregulation of VEGF production by tumour cells [45]. It is interesting to note the correlation between the reduction in β-catenin levels and the observed decrease in FSCN1, which is transcriptionally regulated by β-catenin in tumour cells [38]

One of the main causes of therapeutic inefficiency in cancer patients is drug resistance, which in most cases has features of MDR, with an insensitivity of tumour cells to multiple drugs [46]. To infer the ability of our compound to enhance the antitumor effect of currently adopted chemotherapeutic drugs in melanoma treatment [47], we assessed its combination potential with vemurafenib, dacarbazine and cisplatin. SLMP53-2 displayed synergistic effects with all tested drugs. In line with the relevance of a functional p53 pathway for the effectiveness of many chemotherapeutic agents, the calculated D.R.I. indicated that SLMP53-2 markedly reduced the effective dose of each chemotherapeutic agent. Further supporting the predictive clinical impact of multitargeting p53 and MAPK pathways, a promising C.I. was obtained when combining SLMP53-2 with vemurafenib in a 3D spheroid model of melanoma cells. This strategy was revealed to be particularly relevant in a context of acquired resistance to BRAF-targeting therapy due to reactivation of the MAPK pathway or an exacerbation of the PI3K-AKT pathway to compensate for the inhibition of MAPK pathway [26,27]. Additionally, the loss of expression of PTEN can be also found in some melanomas, corroborating the compensatory phenomenon underlying developed resistance by MAPK-targeting therapy [27,48]. It is also noteworthy that parental and Vem-res melanoma cells did not develop (cross)resistance to SLMP53-2. Importantly, SLMP53-2 enhanced the sensitivity of Vem-res cells to vemurafenib, potentially due to an inhibition of the PI3K-AKT pathway through an enhancement of PTEN expression and downregulation of p-AKT levels. In accordance with this, SLMP53-2 downregulated BCL-2 and MDR-1 protein levels in Vem-res cells treated with SLMP53-2.

SLMP53-2 also displayed in vivo antitumor activity in a xenograft mouse model, suppressing the growth of human melanoma tumours through inhibition of cell proliferation and stimulation of cell death without interfering with body and organ weight of animals. The inhibition of EMT was also confirmed in melanoma tumours treated with SLMP53-2. Since only one melanoma cell line was tested in vivo, in future work, it would be important to further validate these results in other melanoma tumours, particularly expressing mutp53. However, it must be reinforced that a potent antitumor activity of SLMP53-2, with a favourable toxicological profile, was also previously demonstrated in a xenograft mouse of human hepatocellular carcinoma cells [19].

In conclusion, melanoma is a highly metastatic disease with a frequent resistance profile to a broad panel of drugs. The continuous increase in melanoma incidence in western countries and its high clinical aggressiveness have made the development of more effective therapeutic options against melanoma necessary. This work discloses the p53-activating agent SLMP53-2 with encouraging therapeutic potential in melanoma, either as a single agent or in combination regimens. Notably, in addition to its promising effect on melanoma proliferation, SLMP53-2 also revealed great potential against metastatic melanoma and counteracted melanoma resistance to clinically used therapeutic agents. It is still worth noting that in addition to the activation of wtp53, our previous studies have demonstrated the ability of SLMP53-2 to also reactivate wt-like function to mutp53. Despite its lower frequency in melanoma, the appearance of mutp53 can also occur as a genetic, environmental (namely induced by ultraviolet radiation), or even as a consequence of several cycles of treatment [49]. Whether alone or in combination therapy, it became evident that SLMP53-2 is able to interfere with relevant molecular players associated with apoptosis (p53, BAX, KILLER, PUMA, BCL-2, BCL-XL), cell cycle (p21, GADD45, cyclin D1), metastasis (E-cadherin, N-cadherin, vimentin, MMP-2, β-catenin, NRAS, TLR4, TWIST, SLUG, FSCN1), angiogenesis (VEGF), and chemoresistance (MDM2, PTEN, P-ERK, P-AKT, MDR1, BCL-2, BCL-XL). The impact of SLMP53-2 on all these key molecules allows us to predict great clinical outcomes for this compound, particularly by improving overall survival, disease-free survival, and postprogression survival of melanoma patients at different stages of the disease.

## 4. Materials and Methods

### 4.1. Compounds

Tryptophanol-derived oxazoloisoindolinone SLMP53-2 was prepared using a previously described method [19,50].

Cisplatin was purchased from Enzo Life Science (Taper, Sintra, Portugal), vemurafenib from Santa Cruz Biotecnology and Dacarbazine from Sigma-Aldrich (Oeiras, Portugal). All tested compounds were dissolved in DMSO (Sigma-Aldrich) except cisplatin, which was dissolved in saline. In all experiments, the correspondent solvent was included as control in a concentration range that did not affect cell proliferation (maximum concentration used 0.5%).

### 4.2. Human Cell Lines and Growth Conditions

Human melanoma A375 (CLS Cat# 300110/p852_A-375, RRID:CVCL_0132) and SK-MEL-5 (CLS Cat# 300157/p634_SK-MEL-5, RRID:CVCL_0527) cells were purchased from CLS Cell lines service (Eppelheim, Germany). G361 and MEWO human melanoma cells were kindly provided by Dr. Paula Soares (i3S, Porto, Portugal). The tumour cells A375, SK-MEL-5 and MEWO were cultured in RPMI-1640 medium with UltraGlutamine (Lonza, VWR, Carnaxide, Portugal) and G361 cells were cultured in McCoy’s 5A Medium (Lonza). The culture mediums were supplemented with 10% FBS (Gibco, Alfagene, Lisboa, Portugal). Cells were maintained in a humidified incubator at 37 °C with 5% CO_2_. Routine testing for Mycoplasma was performed using the MycoAlertTM PLUS detection kit (Lonza). Additional details about melanoma cell lines can be found in Table S1.

### 4.3. Sulforhodamine B (SRB) Assay

In total, 4.5 x 10^3^ A375 or A375 cells/well, which were resistant to vemurafenib (Vem-res), and 5.0 × 10^3^ SK-MEL-5 cells/well were seeded in 96-well plates and allowed to adhere for 24 h. Cells were then treated with serial dilutions of the compound for an additional 48 h incubation period. Effects on cell proliferation were measured by SRB assay, as described in [51]. IC_50_ values were determined for the tested cell lines using the GraphPad Prism software version 7.0 (RRID:SCR_002798, La Jolla, CA, USA).

### 4.4. Colony Formation Assay

Totals of 5.0 × 10^2^ A375 or MEWO cells/well, 2.0 × 10^3^ SK-MEL-5 cells/well and 7.0 × 10^2^ G361 cells/well were seeded in six-well plates and treated at the seeding time with a range of concentrations of SLMP53-2 for 11 days. Formed colonies were fixed with 10% methanol and 10% acetic acid for 10 min and then stained with 0.5% crystal violet (Sigma-Aldrich) in 1:1 methanol/H_2_O for 15 min. Colonies containing more than 20 cells were counted.

### 4.5. Cell Cycle and Apoptosis Analyses

The analyses were performed as described in [51]. Particularly, 1.2 x 10^5^ A375 cells/well were seeded in 6-well plates and allowed to adhere overnight, followed by treatment with 12 μM SLMP53-2 for 48 h (cell cycle) or 72 h (apoptosis). For cell cycle analysis, cells were stained with propidium iodide (Sigma-Aldrich) and were analysed by flow cytometry; cell cycle phases were identified and quantified using the FlowJo X 10.0.7 Software (RRID:SCR_008520, Treestar, Ashland, OR, USA). For apoptosis, cells were stained using the Annexin V-FITC Apoptosis Detection Kit I from BD Biosciences (Enzifarma, Porto, Portugal), according to the manufacturer’s instructions. The Accuri^TM^ C6 flow cytometer and the BD Accuri C6 software (RRID:SCR_014422, BD Biosciences) were used.

### 4.6. Western Blot Analysis

In total, 1.2 × 10^5^ A375 or A375 Vem-res cells/well and 1.5 × 10^5^ SK-MEL-5 cells/well were seeded in six-well plates for 24 h, followed by treatment with 6 and 12 μM (for A375 cells), 10 and 20 μM (for SK-MEL-5 cells) and 2 μM SLMP53-2 (for A375 Vem-res cells) for the indicated treatment periods. In particular, protein extracts were quantified using the Bradford reagent (Sigma-Aldrich). Proteins were run in SDS-PAGE and then transferred to a Whatman nitrocellulose membrane from Protan (VWR). Membranes were blocked in 5% milk or 5% bovine serum albumin (BSA; for phosphorylation evaluation) and labelled with specific primary antibodies followed by HRP-conjugated secondary antibodies (described in Supplementary Materials, Table S2). GAPDH was used as loading control. The signal was detected with the ECL Amersham kit from GE Healthcare (VWR). For signal detection the ChemiDoc™ MP Imaging System from Bio-Rad Laboratories (Amadora, Portugal) was used. Band intensities were quantified using the Image Lab software version 5.2.1. (RRID:SCR_014210, Bio-Rad Laboratories). Signal intensity is relative to the respective loading and normalized to control (DMSO), set as 1. Whole-blot images and the corresponding quantification are provided in Supplementary Materials (Figure S6 and Table S3).

### 4.7. Transfection of p53 siRNA

A375 cells were seeded in six-well plates and allowed to grow until 50% confluence. Thereafter, cells were transfected with 100 nM siRNAs against p53 (SMARTpool p53) and nonspecific siRNAs (nontargeting pool), both from Thermo Scientific (Bioportugal, Porto, Portugal), using Lipofectamine 2000 (Invitrogen, Alfagene, Lisboa, Portugal), according to the manufacturer’s instructions. After 24 h of transfection, 1.2 × 10^5^ cells/well of control and transfected cells were seeded in six-well plates and immediately treated with a range of concentrations of SLMP53-2. For control of the transfection efficiency, cells were harvested for Western blot analysis of p53 expression levels, as described in Section 4.6. 

### 4.8. RNA Extraction and RT-qPCR

In total, 1.2 × 10^5^ A375 cells/well were seeded in six-well plates for 24 h, followed by treatment with 6 and 12 μM SLMP53-2 for 24 h. Total RNA was extracted from the cells using the Illustra^TM^ RNAspin Mini RNA Isolation Kit (GE Healthcare, Enzymatic, Loures, Portugal). For cDNA synthesis, 1 μg of RNA was used with the NZY M-MuLV Reverse Transcriptase from Nzytech (RRID:SCR_016772, Lisboa, Portugal) in 20 μL final volume following the manufacturer’s instructions. RT-qPCR assays were performed in a 96-well plate on a Real-Time PCR Detection System (Bio-Rad, version 3.1), starting with 16.5 ng of cDNA. The NZY qPCR Green Master Mix (Nzytech) and specific forward and reverse primers for *MDM2* (F-GGCCTGCTTTACATGTGCAA, R-GCACAATCATTTGAATTGGTTGTC), *CDKN1A* (p21; F-CTGGAGACTCTCAGGGTCGAA, R-GATTAGGGCTTCCTCTTGGAG), *TNFRSF10B* (KILLER; F-TGACTCATCTCAGAAATGTCAATTCTTA, R-GGACACAAGAAGAAAACCTTAATGC), *BAX* (F-CCTGGAGGGTCCTGTACAATCT, R-GCACCTAATTGGGCTCCATCT) from Stabvida (Caparica, Portugal) and *TP53* (p53; F-CTCTGACTGTACCACCATCCACTA, R-GAGTTCCAAGGCCTCATTCAGCTC) from Eurofins (MWG, Milan, Italy) were used; *GAPDH* was used as a reference gene.

### 4.9. Cycloheximide (CHX) Assay

A375 cells were seeded in 6-well plates at 1.2 × 10^5^ cells/well for 24 h, followed by 24 h treatment with 12 μM SLMP53-2 or solvent. After this, cells were treated with 150 μg/mL CHX (Sigma-Aldrich) for 0, 0.5, 1, 1.5 and 2 h. p53 protein expression was detected by Western blot as described in Section 2.5; GAPDH was used as loading control.

### 4.10. Coimmunoprecipitation (Co-IP) Assay

For the Co-IP assay, the Pierce Classic Magnetic IP and Co-IP Kit from Thermo Scientific (Dagma, Carcavelos, Portugal) were used. A total of 5.0 × 10^5^/flask A375 cells were treated with 12 and 18 μM SLMP53-2 for 4 h; after cell lysis and protein lysate separation, 300 µg of total protein was incubated with 10 µL of mouse monoclonal anti-p53 (DO-1) or mouse immunoglobulin G (IgG, negative control) from Santa Cruz Biotechnology (Frilabo, Porto, Portugal), overnight at 4 °C. The immunoprecipitation of the immunocomplexes was performed using magnetic beads. The Western blot analysis was performed, as in Section 4.6, for detection of p53 and MDM2 in whole-cell lysate (input) and in immunoprecipitated proteins. GAPDH was used as loading control.

### 4.11. MicroRNA (miRNA) Analysis

A375 cells were seeded in 6-well plates at 1.2 × 10^5^ cells/well for 24 h to adhere, followed by treatment with SLMP53-2. Total RNA was extracted using TRIzol^TM^ reagent (Invitrogen, Carlsbad, CA, USA) according to the manufacturer’s instructions. RNA concentration and purity were measured in NanoDrop™ 1000 (Thermo Fisher Scientific, Waltham, MA, USA). RNA integrity was assessed by gel electrophoresis. miRNA levels were evaluated using TaqMan miRNA assays (Applied Biosystems, Foster City, CA, USA). cDNA was synthesized (MyCycler Thermal Cycler, Bio-Rad) using RNA, TaqMan MicroRNA Reverse Transcription Kit (Applied Biosystems) and gene specific stem-loop Reverse Transcription primers (Applied Biosystems). qPCR reactions were performed in a CFX96 Touch™ Real-Time PCR Detection System (Bio-Rad) using cDNA, hsa-miR-145, hsa-miR-23a, or small nuclear RNA U6 (snRNA U6) TaqMan probes (Applied Biosystems) and SsoAdvanced™ Universal Probes Supermix (Bio-Rad, Hercules, CA, USA). snRNA U6 was used as the reference gene. Relative expression levels were calculated using the quantification cycle (Cq) method according to MIQE guidelines [52].

### 4.12. Generation of Melanoma Spheroids

A375 cells were resuspended in RPMI-1640 culture medium containing 10% FBS. In total, 6 x 10^2^ A375 cells/well were plated in 96-well plates coated with 1% agarose (Sigma-Aldrich). At the seeding time, cells were treated with a concentration range of SLMP53-2 and allowed to growth for 10 days. In another experimental condition, after seeding, spheroids were allowed to grow for 3 days and then treated with SLMP53-2 for 8 days. Fresh medium with drugs was added to the wells each two days. To evaluate the synergistic effect of SLMP53-2 with vemurafenib in spheroids development, 3-day old melanoma spheroids were treated with 2 μM SLMP53-2 and/or 0.027 μM vemurafenib, for an additional 8 days. Fresh medium with the drugs was added to the wells each two days.

Spheroids were photographed using an inverted Nikon TE 2000-U microscope from Nikon Instruments Inc. (Izasa, Carnaxide, Portugal) at ×100 magnification with a DXM1200F digital camera and NIS-Elements microscope imaging software (RRID:SCR_014329, Nikon Instruments Inc.). Determination of spheroid diameter was performed using ImageJ software (v1.8.0, RRID:SCR_003070, Madison, WI, USA) [53].

### 4.13. Combination Therapy Assay

For the assessment of synergistic effects of SLMP53-2 with known chemotherapeutic agents, A375 cells were treated with 2 μM SLMP53-2 and/or increasing concentrations of vemurafenib (0.03–0.5 μM), dacarbazin (0.25–4 μM) and cisplatin (0.3–5 μM) for 48 h. The SRB assay was used to assess the effect of the combined treatments on cell proliferation. For each combination, the combination index (C.I.) and the dose reduction index (D.R.I.) values were calculated using the CompuSyn Software version 1.0 (ComboSyn, Inc., Paramus, NJ, USA) according to the following equation: CI = (D_)1_/(D_x_)_1_ + (D)_2_/(D_x_)_2_, where the numerators (D)_1_ and (D)_2_ are the concentrations of each drug in the combination [(D)_1_ + (D)_2_] that inhibit x%, and the denominators (D_x_)_1_ and (D_x_)_2_ are the concentrations of drug one and two alone that inhibit x%; D.R.I. measures how much the dose of a drug may be reduced in synergistic combination compared to the dose of each drug alone; C.I. values < 1, 1 < C.I. < 1.1 and > 1.1 indicate synergistic, additive and antagonistic effects, respectively [54].

For the 3D spheroid model, the area of melanoma spheroids was assessed using the following equation: A = πab, where (a) corresponds to the major axis and (b) to the minor. The synergistic effect was determined using the additive model: a positive drug combination effect occurs when the observed combination effect (E_AB_) is greater than the expected additive effect given by the sum of the individual effects (E_A_ + E_B_). The C.I. was calculated as described [55,56]: C.I. = (EA + EB)/EAB.

### 4.14. Establishment of Vemurafenib-Resistant Cells

To generate A375 cells resistant to vemurafenib (Res-vem), cells were exposed to several rounds of selection with increasing concentrations of this drug, as previously reported [57,58], starting at the IC_50_ value, and using concentrations 1.5-fold higher in each round until a maximum of 5.0 μM. Vemurafenib was added to culture medium for 24 h, followed by a recovery period of two days in fresh medium without treatment. For the maintenance of the resistance, the Res-vem cells were kept in the presence of vemurafenib at the maximum concentration used to induce resistance and grown in medium without drug for 3 to 4 days before experiments. The same passage number of both parental and resistant cells was used in the experiments. The IC_50_ values of SLMP53-2 and vemurafenib, in parental and Res-vem A375 cells, were determined by SRB assay.

### 4.15. Acquired Resistance Studies

A375 cells were exposed to six rounds of treatments with increasing concentrations of SLMP53-2 (6, 9, 12, 18, 24 and 30 μM). Compound was added to culture medium for 24 h, followed by a recovery time of two days, with fresh medium without treatment. Cells were harvested, seeded and treated twice for each concentration (one round). At the end of each round, IC_50_ values were determined by SRB assay after 48 h treatment. The passage number for both control and surviving resistant cells was the same for each round.

### 4.16. In Vitro Migration and Invasion Assays

For tumour cell migration analysis, both the wound-healing assay and the QCM 24-Well Fluorimetric Chemotaxis Cell Migration Kit (8 μm) from Merck Millipore (Taper) were used as described [59]. Briefly, for the wound-healing assay, 5 × 10^5^ A375 cells/well and 7 × 10^5^ SK-MEL-5 cells/well were grown to confluence in six-well plates, and a fixed-width wound was created in the cell monolayer using a sterile 10 uL micropipette tip. Cells were treated with SLMP53-2 at 2 (for A375 cells) and 4 μM (for SK-MEL-5 cells); images of the wound were captured at different time points using an inverted Nikon TE 2000-U microscope from Nikon Instruments Inc. (Izasa) at 100× magnification with a DXM1200F digital camera (Nikon Instruments Inc.) and NIS-Elements microscope imaging software (version 4; Nikon Instruments Inc.). For calculation of the wound closure, the subtraction of the “wound” area (measured using ImageJ Software) at the indicated time point of treatment to the “wound” area at the starting point was made. For the chemotaxis cell migration assay and fluorimetric cell invasion assay, 1.2 × 10^5^ A375 and SK-MEL-5 cells (cultured in serum-free medium for 24 h) were prepared in serum-free medium for each tested condition. Melanoma cells were treated with SLMP53-2 at 2 (for A375 cells) and 4 μM (for SK-MEL-5 cells). The prepared cell suspensions were distributed in 24-well plates (300 μL per insert), followed by an addition of 500 μL medium containing 10% FBS to the lower chamber. After 24 h, cells that migrated or invaded through the ECMatrix layer (with 8 μm pore membranes) were eluted, lysed and stained with a green-fluorescence dye that binds to cellular nucleic acids. The number of migrating/invading cells was proportional to the fluorescence signal measured using the Bio-Tek Synergy HT plate reader (Izasa), at 480/520 nm (ex/em).

### 4.17. Measurement of Extracellular Lactate

Using the Lactate-Glo™ assay kit (Promega, VWR), the lactate levels exported by A375 and SK-MEL-5 cells to the culture medium were determined according to the manufacturer’s instructions. Briefly, 1.2 × 10^5^ A375 cells/well and 1.5 × 10^5^ SK-MEL-5 cells/well were seeded in six-well plates, followed by treatment with a concentration range of SLMP53-2. After 8 h treatment, the culture medium was collected and diluted in phosphate saline buffer (1:200). Then, 50 μL was transferred to a 96-well assay plate and 50 μL of Lactate Detection Reagent Mix was added. The plate was kept 60 min at room temperature followed by luminescence assessment using the Bio-Tek Synergy HT plate reader (Izasa).

### 4.18. In Vivo Antitumour Assay

The animal studies reported are in compliance with the ARRIVE guidelines [60]. All animals were housed in polycarbonate cages (two to six per cage) and kept on a 12 h light/dark cycle. Food and water were given ad libitum. Studies were reviewed by the Animal Ethics Committee and Animal Welfare Body of the i3S (reference 2016/22), authorized by the national authority *Direção Geral de Alimentação e Veterinária* (DGAV reference 0421/000/000/2017). Animal studies were performed using the C57BL/6-Rag2^−/−^IL2rg^−/−^ mouse model (negative for B and T cells to allow tumour growth; other mouse strains with less compromised immune systems led to a marked delayed in tumour growth kinetic, which showed to be incompatible with the intended treatment regimen), generously provided by Prof. James Di Santo (Institute Pasteur, Paris, France). In total, 9.0 × 10^6^ A375 cells (in PBS/Matrigel 1:1; Corning, Enzifarma, Porto, Portugal) were subcutaneously inoculated into the right flank of male and female mice of 7 to 11 weeks (randomized into two groups, three male and four female mice used in each group). Tumour volume was routinely measured using a calliper and the formula (a × b^2^)/2 (where a and b represent the longest and shortest tumour axis, respectively). When tumour size reached approximately 100 mm^3^ (7 days after implantation), mice were randomized into two experimental groups, with equal representation of genders between experimental groups to avoid gender bias (three males and four female mice per group). Twice-weekly intraperitoneal injections of 50 mg∙kg^−1^ SLMP53-2 or vehicle (seven animals per group) were started for tumours with approximately 100 mm^3^ (7 days after implantation). Six administrations were performed with continuous monitoring of tumour volume (endpoint set at 2000 mm^3^), animal weight variation (endpoint set at 10% of weight loss), physical appearance and signs of morbidity. At the end of treatment, animals were anaesthetized with a volatile anaesthetic (5% isoflurane, 1L/min oxygen) and sacrificed by cervical dislocation.

### 4.19. Immunohistochemical (IHC) Analysis

Tumour tissues from five animals per group were analysed by IHC. Tissues were fixed in 10% formalin, embedded in paraffin, sectioned at 4 μm, and stained with haematoxylin and eosin (H&E) or antibodies as described [59]. Briefly, antigen retrieval was performed by boiling the sections for 20 min in citrate buffer (pH 6.0). Antibodies used are listed in (Supporting information, Table S2). Immunostaining was performed using the UltraVision Quanto Detection System HRP DAB Kit from Lab Vision Thermo Scientific (Taper) according to the manufacturer’s instructions. Evaluation of 3,3′-diaminobenzidine (DAB) intensity and quantification of stained cells were performed using ImageJ software version 1.8.0. (Madison, WI, USA). Images were obtained using an Eclipse E400 fluorescence microscope (Nikon) with ×200 magnification, with a Digital Sight camera system (Nikon DS-5Mc) and software Nikon ACT-2U (Izasa). The quantification of DAB intensity in cytoplasmic staining was performed as described in [61]. TUNEL assay was performed using the In situ Cell Death.

Detection Kit Fluorescein (Roche, Ingrenor, Porto, Portugal) was used according to the manufacturer’s instructions. Tissues were counterstained with DAPI (0.1 μg/mL). For quantification of stained cells with nuclear staining, ImageJ was also used to count total positive labelled cells. Images were obtained using an Eclipse E400 fluorescence microscope (Nikon) with ×200 magnification, with a Digital Sight camera system (Nikon DS-5Mc) and software Nikon ACT-2U (Izasa), and five random fields were selected and acquired for analysis.

### 4.20. Data and Statistical Analysis

Data are presented as mean ± SEM of “n” samples, where “n” refers to independent experiments not replicates. Values of “n” and number of technical replicates, if performed, are given in figure legends. Where replicates were used, their values were averaged to provide a single value to the data set. Data analyses were carried out using GraphPad Prism Software version 7.0 (La Jolla). All assays with five or more independent experiments were subjected to statistical analysis. In some data sets, log transformation was carried out to generate Gaussian-distributed data set. Normalization was carried out to control unwanted sources of variation, and data analysis was performed setting controls (DMSO or nontreated cells) as 100% or as one for comparison purposes. For comparison of two groups, unpaired Student’s *t*-test was used. For comparison of multiple groups, statistical analysis relative to controls was performed using one-way or two-way ANOVA followed by post hoc Tukey’s, Sidak’s or Dunnet’s multiple comparison tests.

## 5. Conclusions

SLMP53-2 may represent a valuable contribution to the advancement of personalized melanoma therapy in comparison to other p53–MDM2 interaction inhibitors, such as nutlin-3a, which is only effective against wtp53-expressing tumours. It may also be the starting point for the development of improved pharmacological agents against advanced melanomas that still lack effective therapeutic options.

## 6. Patents

The compound SLMP53-2 is protected under an international patent (European patent EP3013833 and US patent 20160347765).

## Data Availability

Data presented in this study are available in the article or Supplementary Files or upon request to the corresponding author.

## Supplementary Materials

The following are available online at https://www.mdpi.com/article/10.3390/cancers13071648/s1, Figure S1. Endogenous p53 expression levels in melanoma cell lines. Figure S2. Concentration–response curves for SLMP53-2 in A375 (A–C) and SK-MEL-5 (D,E) cells. Figure S3. SLMP53-2 regulates the protein expression levels of p53 and its transcriptional targets in SK-MEL-5 and MEWO cells. Figure S4. SLMP53-2 does not enhance wtp53 binding to HSP70 or HSP90 in A375 melanoma cells. Figure S5. Melanoma cells develop resistance to vemurafenib. Figure S6. Whole-blot images. Table S1. p53 and BRAF status in melanoma cell lines: IC50 values of SLMP53-2. Table S2. List of antibodies used in Western blot (WB) and immunohistochemistry (IHQ). Table S3. Quantification of Western blots.
