# Supplementary material for: Targeting p53 for Melanoma Treatment: Counteracting Tumour Proliferation, Dissemination and Therapeutic Resistance
Source: Cancers (Basel). 2021 Apr 1;13(7):1648. doi: 10.3390/cancers13071648 (PMC8037490; doi:10.3390/cancers13071648)
Supplement: Supplementary file 1 [file cancers-13-01648-s001.pdf]

cancers

MDPI

# Supplementary Materials: Targeting p53 for Melanoma Treatment: Counteracting Tumour Proliferation, Dissemination and Therapeutic Resistance

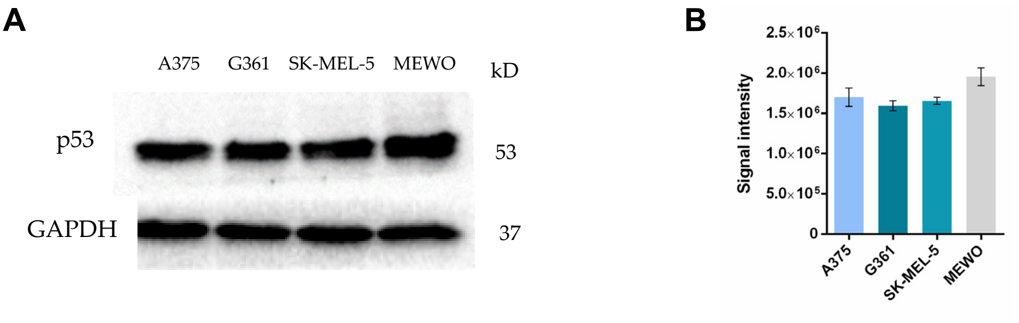


**Figure S1.** Endogenous p53 expression levels in melanoma cell lines. **(A)** p53 expression levels were evaluated in melanoma cells expressing wtp53 (A375, SK-MEL-5, G361) or mutp53 (MEWO), by western blot analysis. **(B)** Quantification of p53 protein expression levels; values of signal intensity are plotted; data are mean ± SEM, $n = 5$.

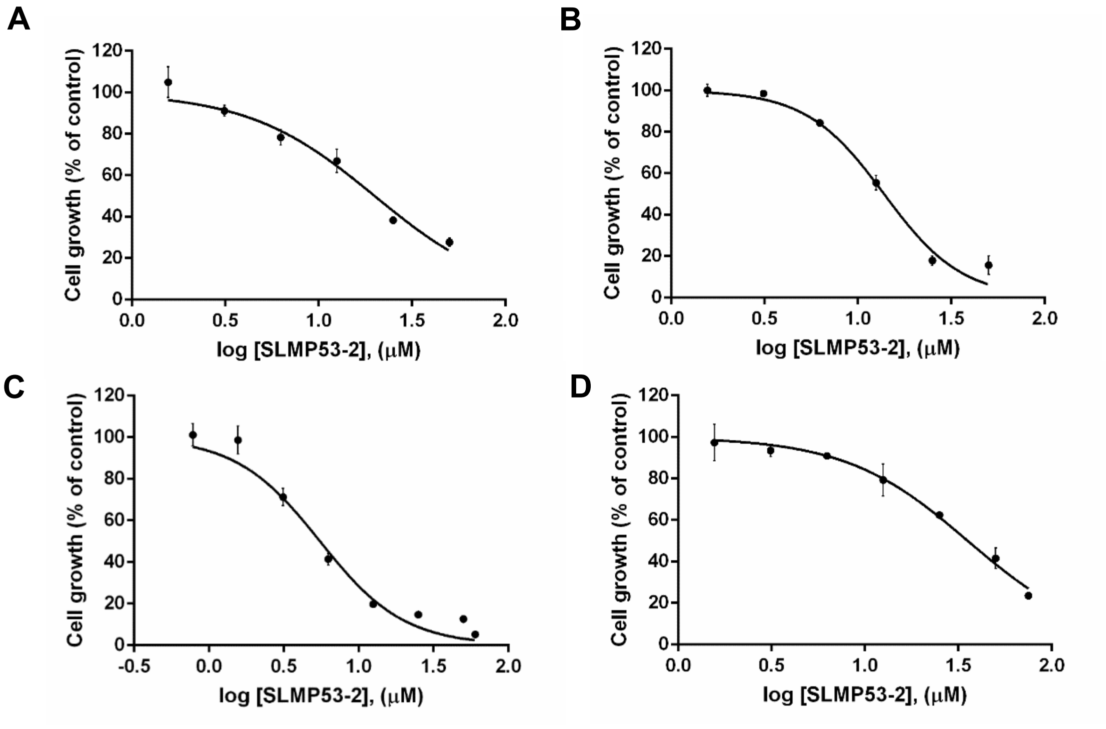

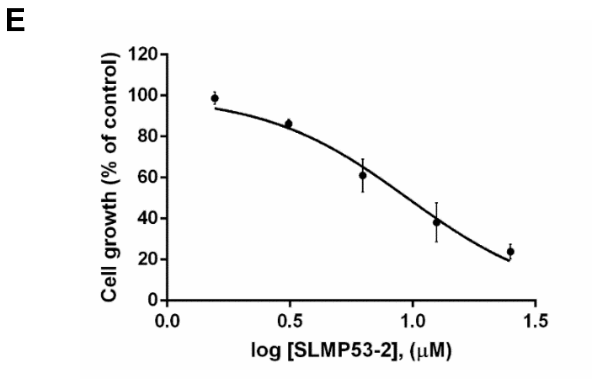


**Figure S2.** Concentration–response curves for SLMP53-2 in A375 **(A–C)** and SK-MEL-5 **(D,E) cells. (A)** SLMP53-2 was tested from 1.56 – 50 μM, for 24 h. **(B)** SLMP53-2 was tested from 1.56 – 50 μM, for 32 h. **(C)** SLMP53-2 was tested from 0.78 – 60 μM, for 48 h. **(D)** SLMP53-2 was tested from 1.56 – 75 μM, for 30 h. **(E)** SLMP53-2 was tested from 1.56 – 25 μM, for 48 h. In **A-E**, data were normalized to DMSO and correspond to mean ± SEM, *n* = 6 (two replicates each).

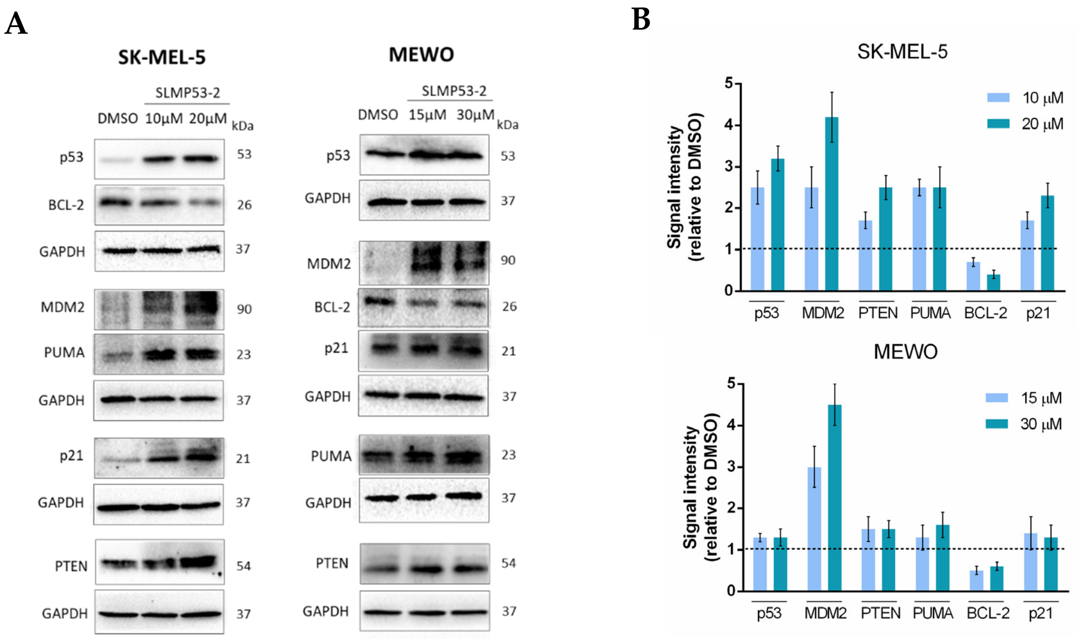


**Figure S3.** SLMP53-2 regulates the protein expression levels of p53 and its transcriptional targets, in SK-MEL-5 and MEWO cells. Melanoma cells were treated with $IC_{50}$ and two-fold $IC_{50}$ SLMP53-2 for 48 h (24 h for p21 in SK-MEL-5) (A) Immunoblots are representative of five independent experiments; GAPDH was used as loading control. (B) Quantification of protein expression levels; values with DMSO were set as 1; data are mean ± SEM, n = 5.

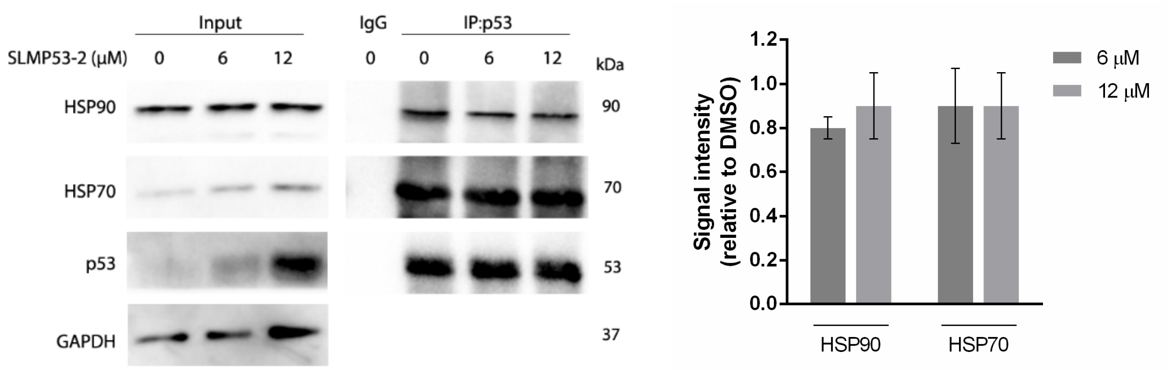


**Figure S4.** SLMP53-2 does not enhance the wtp53 binding to HSP70 or HSP90, in A375 melanoma cells. Co-IP assay was performed in A375 cells treated with SLMP53-2 for 8 h. Quantification of immunoprecipitated protein expression levels; immunoblots are representative of three independent experiments; p53 from IP was used as loading control and DMSO was set as 1; data are mean ± SEM, n = 5.

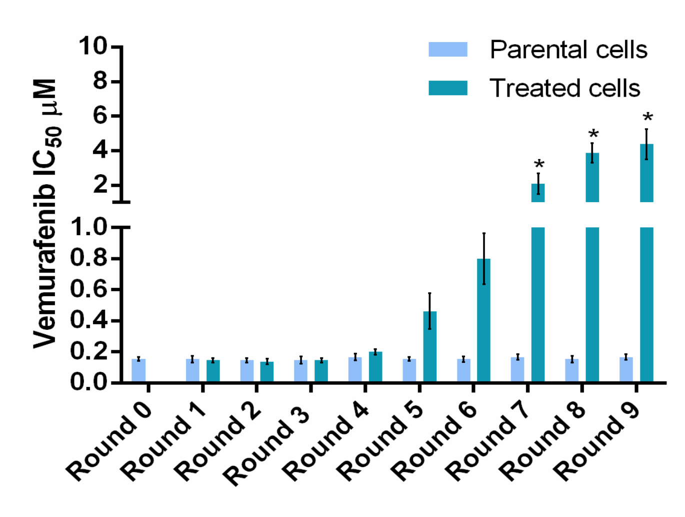


**Figure S5.** Melanoma cells develop resistance to vemurafenib. A375 cells were exposed to increasing concentrations of vemurafenib. $IC_{50}$ values were determined at the end of each round by SRB assay after 48 h treatment. Data were normalized to DMSO and correspond to mean ± SEM, $n = 5$ (two replicates each); values of vemurafenib-treated cells significantly different from parental cells: *$p<0.05$, two-way ANOVA followed by Sidak's test.

**Figure S6.** Whole blot images.

Figure 2A

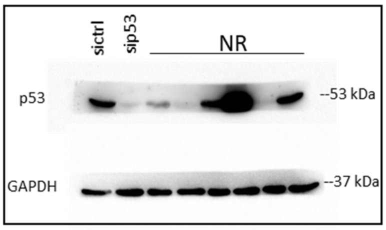


Samples for other non-related works:

NR

Figure 2D

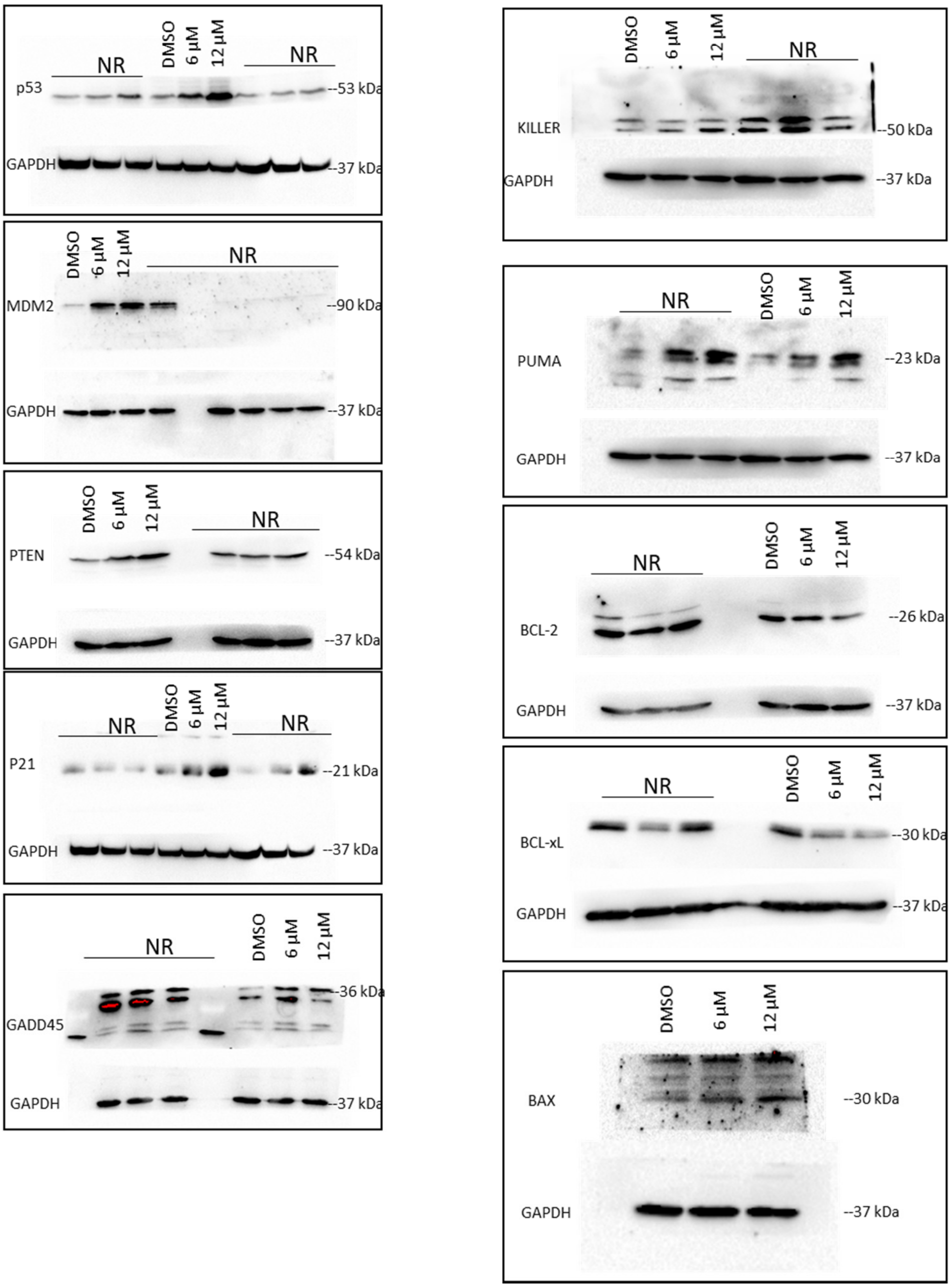

Figure 3A

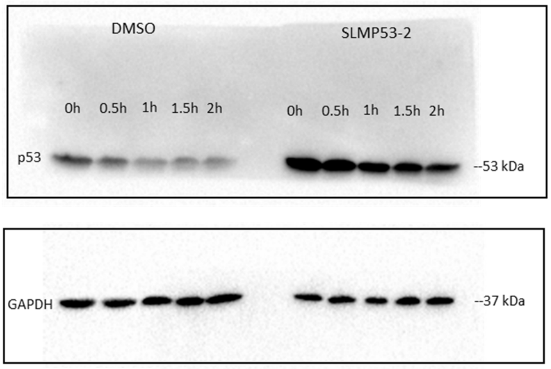


Figure 3C

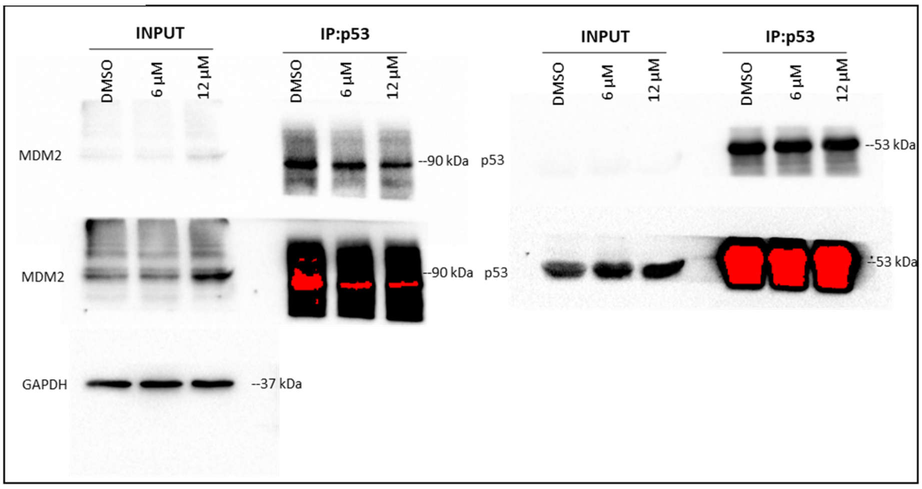


Figure 3F

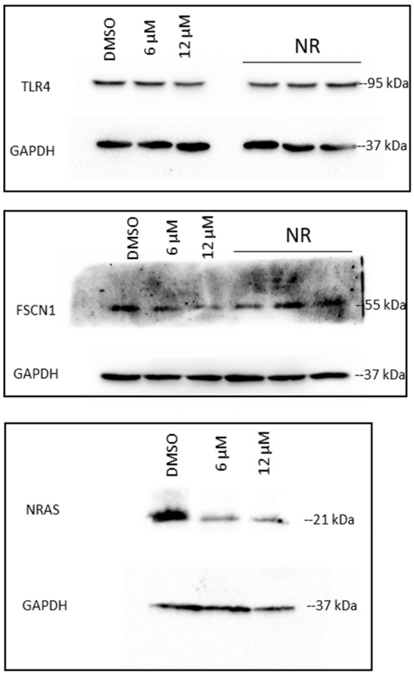


2

Figure 5A

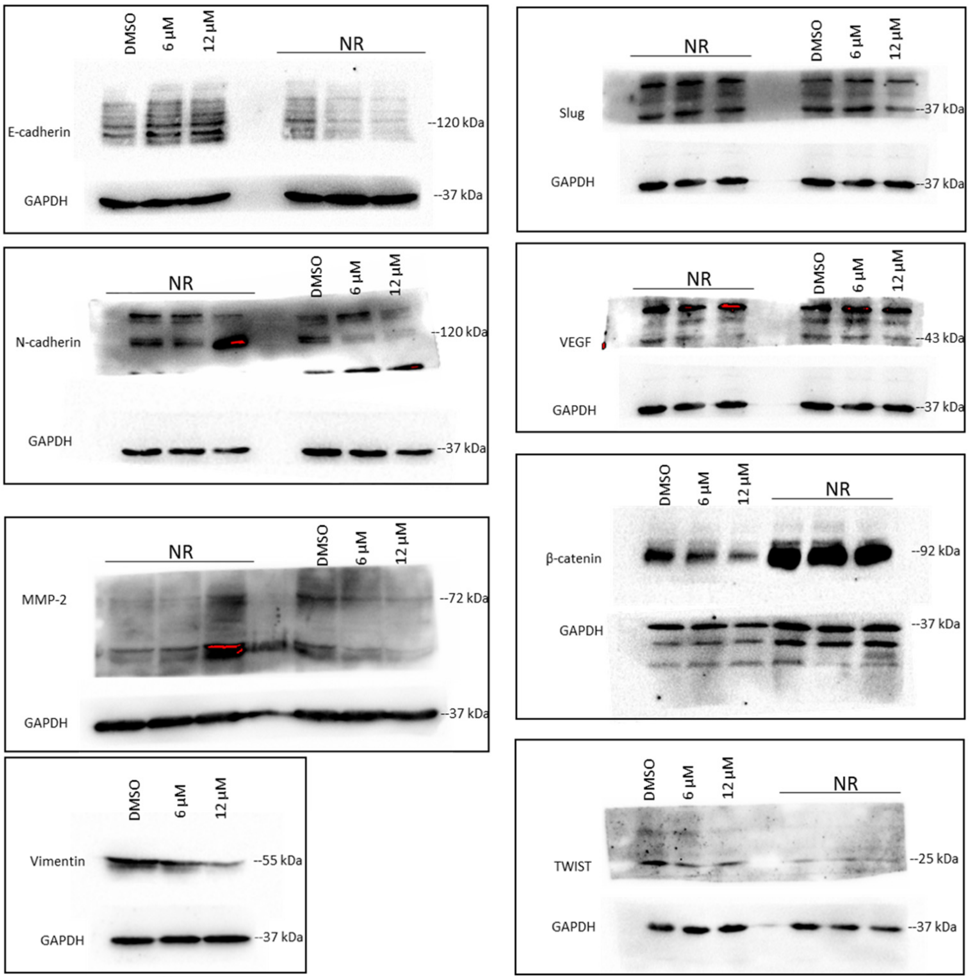


Figure 5C

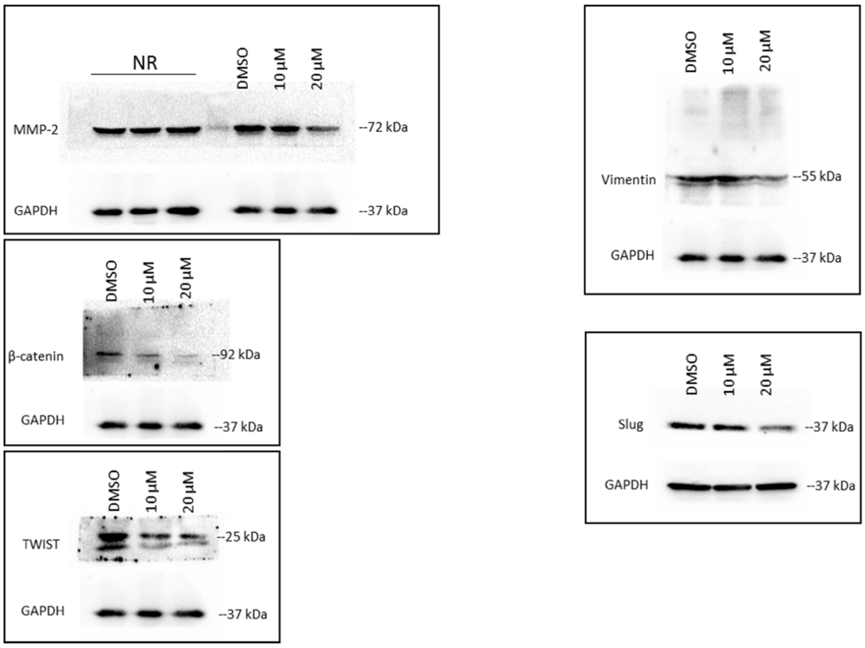


3

Figure 6E

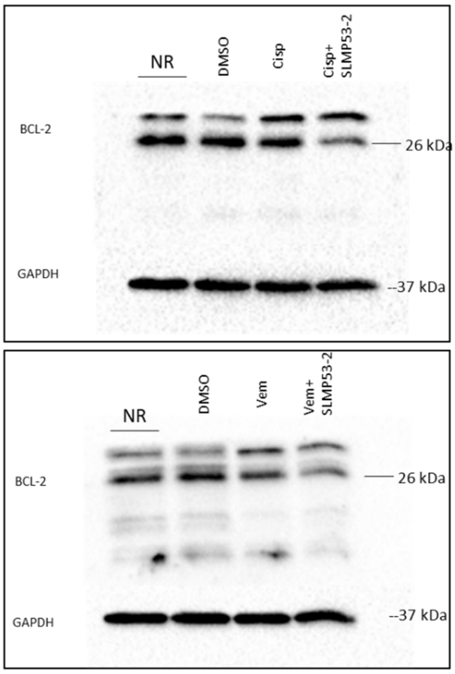


Figure 6H

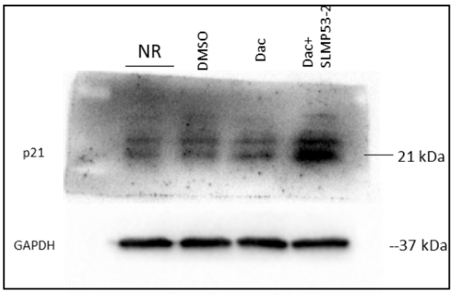

Figure 7D

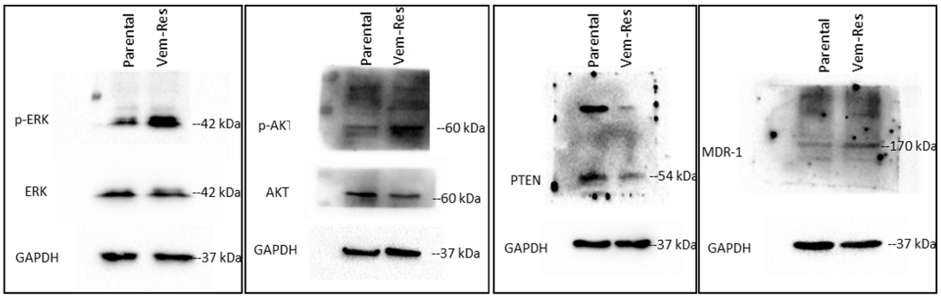


Figure 7I

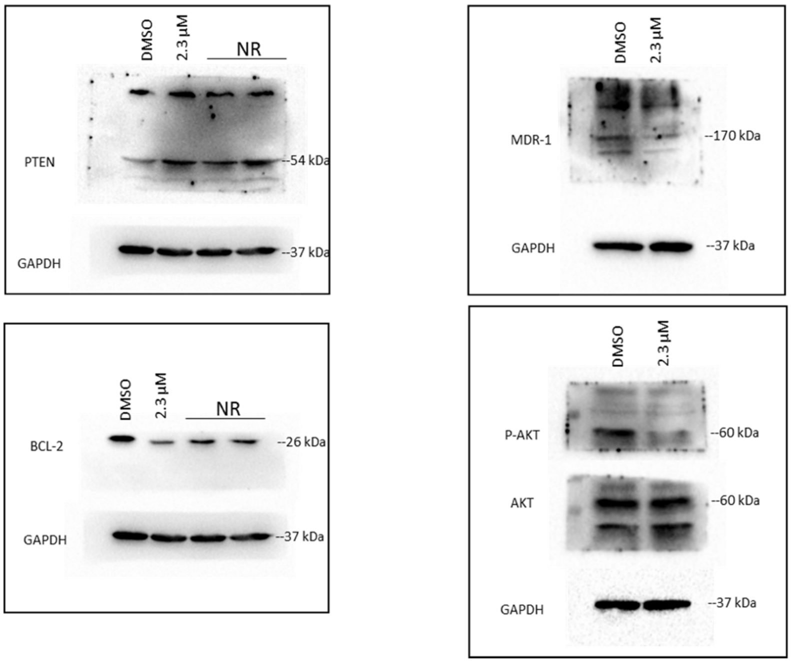


**Table S1.** p53 and BRAF status in melanoma cell lines: $IC_{50}$ values of SLMP53-2.

| Melanoma cells | p53 status | BRAF status | $IC_{50}$ (μM) |
|---|---|---|---|
| **A375** | wt | mutBRAF$^{V600E}$ | 3.5 ± 1.1 |
| **SK-MEL-5** | wt | mutBRAF$^{V600E}$ | 7.6 ± 1.0 |
| **G361** | wt | mutBRAF$^{V600E}$ | 3.5 ± 1.1 |
| **MEWO** | mutp53$^{E258K}$ | wt | 8.5 ± 1.1 |

$IC_{50}$ values of SLMP53-2 were determined by colony formation assay; data are mean ± SEM, n = 5.

**Table S2.** List of antibodies used in western blot (WB) and immunohistochemistry (IHQ).

| Antigen. | Final Dilution | Supplier |
|---|---|---|
| **Primary Antibodies** | | |
| Akt1/2/3 (5C10)<br>Mouse monoclonal | 1:200 | Santa Cruz Biotechnology<br>Cat# sc-81434, RRID:AB_1118808 |
| Bax (6A7)<br>Mouse monoclonal | 1:100 (WB)<br>1:20 (IHQ) | Thermo Scientific<br>Cat# MA5-14003, RRID:AB_10979735) |
| BCL-xL (H-5)<br>Mouse monoclonal | 1:100 | Santa Cruz Biotechnology<br>Cat# sc-8392, RRID:AB_626739 |
| BCL-2 (C-2)<br>Mouse monoclonal | 1:200 | Santa Cruz Biotechnology<br>Cat# sc-7382, RRID:AB_626736 |
| Cyclin D1 (A-12) | 1:100 | Santa Cruz Biotechnology |

| | | |
|---|---|---|
| Mouse monoclonal | | Cat# sc-8396, RRID:AB_627344 |
| E-cadherin (67A4)<br>Mouse monoclonal | 1:100 | Santa Cruz Biotechnology<br>Cat# sc-21791, RRID:AB_626777 |
| ERK 2 (4C11C11C4)<br>Mouse monoclonal | 1:300 | Santa Cruz Biotechnology<br>Cat# sc-65981, RRID:AB_1122620 |
| Fascin 1 (D-10)<br>Mouse monoclonal | 1:200 | Santa Cruz Biotechnology<br>Cat# sc-46675, RRID:AB_627582 |
| GADD45α (C-4)<br>Mouse monoclonal | 1:100 | Santa Cruz Biotechnology<br>Cat# sc-6850, RRID:AB_627653 |
| GAPDH (6C5)<br>Mouse monoclonal | 1:10000 | Santa Cruz Biotechnology<br>Cat# sc-32233, RRID:AB_627679 |
| KILLER/DR5 (D-6)<br>Mouse monoclonal | 1:100 | Santa Cruz Biotechnology<br>Cat# sc-166624, RRID:AB_2204942 |
| Ki-67 (SP6)<br>Rabbit monoclonal | 1:200 | Thermo Fisher Scientific<br>Cat# MA5-14520, RRID:AB_10979488 |
| MDM2 (SMP14)<br>Mouse monoclonal | 1:100 | Santa Cruz Biotechnology<br>Cat# sc-965, RRID:AB_627920 |
| MDR-1 (D-11)<br>Mouse monoclonal | 1:100 | Santa Cruz Biotechnology<br>Cat# sc-55510, RRID:AB_629007 |
| MMP-2 (2C1)<br>Mouse monoclonal | 1:100 | Santa Cruz Biotechnology<br>Cat# sc-13594, RRID:AB_627956 |
| N-cadherin (13A9)<br>Mouse monoclonal | 1:100 | Santa Cruz Biotechnology<br>Cat# sc-59987, RRID:AB_781744 |
| N-Ras (F155)<br>Mouse monoclonal | 1:150 | Santa Cruz Biotechnology<br>Cat# sc-31, RRID:AB_628041 |
| p-Akt1/2/3 (11E6)<br>Mouse monoclonal | 1:100 | Santa Cruz Biotechnology<br>Cat# sc-81433, RRID:AB_1125472 |
| p-ERK (E-4)<br>Mouse monoclonal | 1:200 | Santa Cruz Biotechnology<br>Cat# sc-7383, RRID:AB_627545 |
| PTEN (A2B1)<br>Mouse monoclonal | 1:500 | Santa Cruz Biotechnology<br>Cat# sc-7974, RRID:AB_628187 |
| PUMA (B-6)<br>Mouse monoclonal | 1:50 | Santa Cruz Biotechnology<br>Cat# sc-377015, RRID:AB_2714161 |
| p21 (C-19)<br>Rabbit polyclonal | 1:100 | Santa Cruz Biotechnology<br>Cat# sc-397, RRID:AB_632126 |
| p53 (DO-1)<br>Mouse monoclonal | 1:5000 | Santa Cruz Biotechnology<br>Cat# sc-126, RRID:AB_628082 |
| SLUG (A-7)<br>Mouse monoclonal | 1:200 | Santa Cruz Biotechnology<br>Cat# sc-166476, RRID:AB_2191897 |
| TLR4 (25)<br>Mouse monoclonal | 1:200 | Santa Cruz Biotechnology<br>Cat# sc-293072, RRID:AB_10611320 |
| VEGF<br>Mouse monoclonal | 1:100 | Thermo Scientific<br>Cat# MA1-16629, RRID:AB_2212682 |
| Vimentin (E-5)<br>Mouse monoclonal | 1:1000 (WB)<br>1:200 (IHQ) | Santa Cruz Biotechnology<br>Cat# sc-373717, RRID:AB_10917747 |
| β-catenin (E-5)<br>Mouse monoclonal | 1:500 (WB)<br>1:50 (IHQ) | Santa Cruz Biotechnology<br>Cat# sc-7963, RRID:AB_626807 |
| **Secondary antibodies** | | |
| Anti-mouse<br>HRP-conjugated | 1:5000 | Abcam<br>Cat# ab6789, RRID:AB_955439 |
| Anti-rabbit<br>HRP-conjugated | 1:5000 | Abcam<br>Cat# ab6721, RRID:AB_955447 |

**Table S3.** Quantification of western blots.

| **Figure 2 - A** | | |
|---|---|---|
| | CTRL | Sip53 |
| p53 | 1.00 | 0.07 |

| **Figure 2 - D** | | | |
|---|---|---|---|
| | DMSO | 6 μM | 12 μM |
| p53 | 1.00 | 1.83 | 5.34 |
| MDM2 | 1.00 | 2.20 | 4.25 |
| PTEN | 1.00 | 1.81 | 2.99 |
| p21 | 1.00 | 2.08 | 4.76 |
| Cyclin D1 | 1.00 | 0.50 | 0.32 |
| GADD45 | 1.00 | 3.56 | 4.33 |
| KILLER | 1.00 | 2.47 | 4.31 |
| PUMA | 1.00 | 1.36 | 5.58 |
| BCL-2 | 1.00 | 0.78 | 0.37 |
| BCL-xL | 1.00 | 0.52 | 0.40 |
| BAX | 1.00 | 1.53 | 1.85 |

| Figure 3– A | | | | | |
|---|---|---|---|---|---|
| DMSO | 0h | 0.5h | 1h | 1.5h | 2h |
| p53 | 1.00 | 0.63 | 0.49 | 0.40 | 0.36 |
| SLMP53-2 | 0h | 0.5h | 1h | 1.5h | 2h |
| p53 | 1.00 | 1.01 | 0.87 | 0.76 | 0.59 |

| Figure 3 - C | | | |
|---|---|---|---|
| Input | | IP | |
| p53 | | p53 | |
| DMSO | 1.00 | DMSO | 1.00 |
| 6 μM | 2.07 | 6 μM | 0.98 |
| 12 μM | 3.98 | 12 μM | 1.01 |
| MDM2 | | MDM2 | |
| DMSO | 1.00 | DMSO | 1.00 |
| 6 μM | 1.20 | 6 μM | 0.80 |
| 12 μM | 2.14 | 12 μM | 0.62 |

Figure 3 - G

| | DMSO | 6 μM | 12 μM |
|---|---|---|---|
| TLR4 | 1.00 | 0.80 | 0.44 |
| FSCN1 | 1.00 | 0.78 | 0.45 |
| NRAS | 1.00 | 0.52 | 0.58 |

Figure 5 - A

| | DMSO | 6 μM | 12 μM |
|---|---|---|---|
| E-cadherin | 1.00 | 1.35 | 1.65 |
| N-cadherin | 1.00 | 0.27 | 0.22 |
| Vimentin | 1.00 | 0.54 | 0.37 |
| MMP-2 | 1.00 | 0.49 | 0.43 |
| β-catenin | 1.00 | 0.70 | 0.43 |
| Slug | 1.00 | 0.99 | 0.67 |
| VEGF | 1.00 | 0.58 | 0.44 |
| TWIST | 1.00 | 0.45 | 0.40 |

Figure 5 – C

| | DMSO | 10 μM | 20 μM |
|---|---|---|---|
| Vimentin | 1.00 | 0.99 | 0.60 |
| MMP-2 | 1.00 | 0.75 | 0.31 |
| β-catenin | 1.00 | 0.66 | 0.43 |
| Slug | 1.00 | 0.82 | 0.52 |
| TWIST | 1.00 | 0.72 | 0.23 |

Figure 6 – E

| | DMSO | Cisp | SLMP +Cisp |
|---|---|---|---|
| BCL-2 | 1.00 | 0.8 | 0.40 |
| | DMSO | Vem | SLMP +Vem |
| BCL-2 | 1.00 | 0.8 | 0.6 |

**Figure 6 – H**

| | DMSO | Dac | SLMP +Dac |
|---|---|---|---|
| p21 | 1.00 | 1.5 | 2.90 |

**Figure 7 – D**

| | Parental | Vem-Res |
|---|---|---|
| p-ERK | 1.00 | 2.34 |
| ERK | 1.00 | 0.93 |
| p-AKT | 1.00 | 1.75 |
| AKT | 1.00 | 0.70 |
| PTEN | 1.00 | 0.56 |
| MDR-1 | 1.00 | 1.70 |

Figure 7 – I

| | Parental | Vem-Res |
|---|---|---|
| PTEN | 1.00 | 2.35 |
| BCL-2 | 1.00 | 0.48 |
| MDR-1 | 1.00 | 0.51 |
| p-AKT | 1.00 | 0.54 |
| AKT | 1.00 | 0.94 |
